# Research Progress in Thermal Functional Fibers

**DOI:** 10.3390/ma19010011

**Published:** 2025-12-19

**Authors:** Hui Zheng, Xiao Yang, Chunyang Wang, Yujie Xu, Haisheng Chen, Ting Zhang, Xinghua Zheng

**Affiliations:** 1 Institute of Engineering Thermophysics, Chinese Academy of Sciences, Beijing 100190, China; zhenghui@iet.cn (H.Z.); yangxiao@iet.cn (X.Y.); wangchunyang@iet.cn (C.W.); xuyujie@iet.cn (Y.X.); 2 University of Chinese Academy of Sciences, Beijing 100049, China; 3 Key Laboratory of Long-Duration and Large-Scale Energy Storage, Chinese Academy of Sciences, Beijing 100190, China; 4 Nanjing Institute of Future Energy System, Nanjing 211135, China; 5 University of Chinese Academy of Sciences, Nanjing 211135, China

**Keywords:** fibers, thermal regulation, thermoelectric, photothermal conversion, thermal actuation fiber materials

## Abstract

The utilization and transformation of heat have played pivotal roles in numerous significant stages of human societal evolution and advancement. Recently, more rigorous and precise requirements have been imposed on thermal functional materials for applications including microelectronic device cooling, personal thermal regulation in extreme environments, green building initiatives, flexible wearable electronics, and solar thermal collection. Thermal functional fibers offer advantages such as lightweight construction, versatile functional design, and integrated manufacturing capabilities. By modifying the composition, structure, and fabrication techniques of fibers, control over heat transfer, storage, and conversion processes can be optimized. This review underscores the latest developments in thermal functional fibers, emphasizing high thermal conductivity fibers, thermal insulation fibers, thermal radiation regulation fibers, phase-change thermal storage fibers, thermoelectric fibers, Joule heating fibers, photothermal conversion fibers, thermally actuated fibers, and multifunctional composite fibers. It elucidates how these various fibers enhance thermal performance through innovative material selection, fabrication methods, and structural design. Finally, the review discusses prevailing developmental trends, current challenges, and future directions in the design and fabrication of thermal functional fibers.

## 1. Introduction

Thermal energy, recognized as a vital form of energy, has played an essential role in the development of human society through its acquisition, utilization, and management across various historical periods. In ancient times, fire symbolized heat and was utilized for warmth, cooking, and even metal smelting, marking humanity’s earliest acknowledgment and application of thermal energy. During the Industrial Revolution, the steam engine effectively converted thermal energy into mechanical energy, facilitating its widespread industrial application. In the modern era, rapid societal advancement and technological innovations have opened up new avenues for the utilization and management of thermal energy, while also presenting new challenges. Within the domain of thermal management, considerable focus has been directed towards personal thermal regulation in extreme environments [1], thermal control in electronic devices such as smartphones, vehicles, and aerospace equipment [2,3], and thermal management in built environments [4]. Concerning thermal utilization, extensive research has been conducted in areas such as solar thermal collection [5], seawater desalination [6], and solar thermal power generation [7]. Additionally, notable progress has been achieved in specialized functional applications of heat, including biomedical uses [8], thermal camouflage [9], radiative cooling [10], and information storage and transmission [11].

Fibers are a class of slender solid units characterized by high aspect ratios, minute cross-sectional dimensions, and inherent processability, tensile strength, and flexibility. They can be employed independently or assembled to produce yarns, fabrics, and fiber-reinforced composite materials. In comparison to bulk and thin-film materials, fibers are characterized by attributes such as being lightweight, possessing a high strength-to-weight ratio, and allowing for facile functionalization. The development and application of fibers have a long-standing history. Fibers can be classified into natural fibers and man-made fibers based on their sources. During the early development of human society, Initially, natural fibers—exemplified by silk, cotton, linen, and wool—catalyzed the rapid growth of the textile industry [12]. Subsequently, with the advancement of the chemical industry, artificial fibers [13,14] gradually demonstrated greater advantages in terms of cost and performance. Artificial fibers can be categorized based on their raw materials into regenerated cellulose fibers, regenerated protein fibers, synthetic polymer fibers, and inorganic artificial fibers. Among these, synthetic polymer fibers are produced from petrochemical synthetic polymers such as polyester, polyamide, and polyolefin through various spinning processes. Inorganic artificial fibers, on the other hand, include glass fibers, carbon fibers, ceramic fibers, and metal fibers, among others. Amid ongoing progress in materials science and manufacturing techniques, coupled with increasing demand for multifunctional fibers, innovative composite functional fibers with specialized properties—including electrical conductivity, thermal conductivity, optical transmission, and flame retardancy—have been developed. Among these, thermally functional fibers constitute a significant category, referring to fibers produced via diverse composite spinning methods that exhibit distinctive thermal properties. Examples encompass highly thermally conductive carbon fibers [15], thermally insulating aerogel fibers [16], fibers with photothermal conversion capabilities [17], and composite fibers with multiple integrated functionalities [18]. These thermally functional fibers have demonstrated remarkable performance across various applications, including personal and building thermal management, flexible wearable electronics, biomedical fields, signal transmission, and energy and environmental sectors.

Currently, thermal functional fibers mainly regulate heat through three aspects: heat transfer, heat storage, and heat conversion. Regarding the heat transfer process, it is primarily divided into two methods: high thermal conductivity fibers, which improve heat conduction, convection, and radiation, and thermal insulating fibers, which aim to block heat transfer. For heat storage, phase-change materials are mainly used in heat-storage fibers to provide thermal storage. Concerning heat conversion, it mainly involves thermal functional fibers that enable special functions such as energy supply, refrigeration, and information transmission by converting thermal energy into other forms of energy or vice versa. The functional design of thermal functional fibers is achieved through three main approaches: first, using raw materials with inherently excellent thermal properties; second, designing the fiber’s internal microstructure to achieve specific functionalities; and third, creating multifunctional composite fibers by combining different functional materials through specialized fiber manufacturing methods.

**Figure 1:**
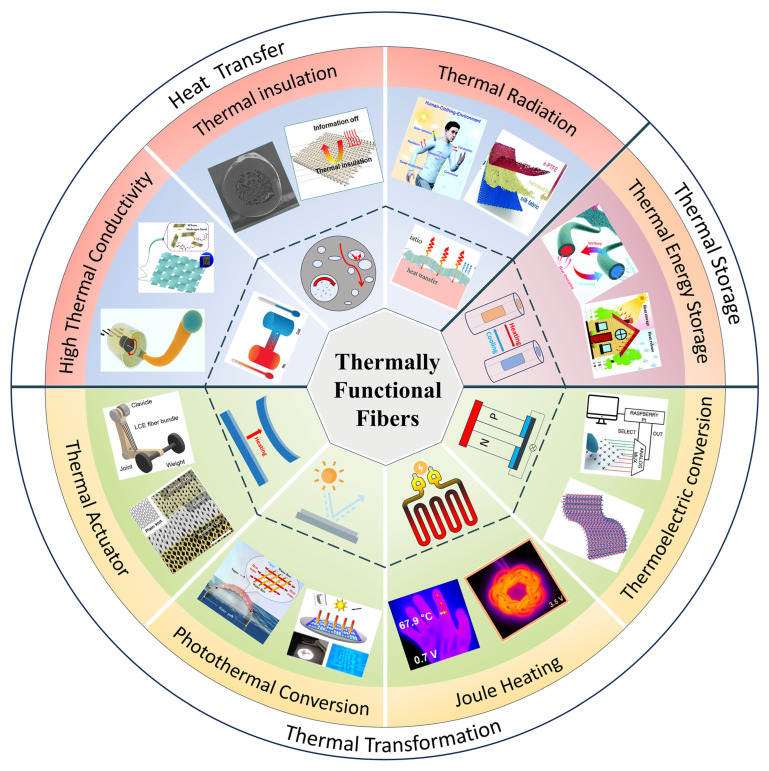
Thermal functional fibers.

This paper reviews the latest advancements in thermal functional fibers. Systematically categorized into heat transfer, heat storage, and heat conversion, it presents the current design strategies, fabrication methods, evaluation metrics, as well as the advantages and limitations of various thermal functional fibers. Finally, it summarizes the developmental status, future prospects, and existing challenges in this field. Figure 1 provides a schematic representation delineating the core principles and application areas of diverse thermal functional fibers.

## 2. Fibers Designed for Manipulating Heat Transfer Processes

Heat transfer describes the process where thermal energy naturally moves from a region of higher to lower temperature under a temperature gradient [19]. The rate of heat transfer influences the distribution of the temperature field within the medium. Heat transfer typically occurs through three main mechanisms: thermal conduction, thermal convection, and thermal radiation. The thermophysical properties of the heat transfer medium can significantly affect the rate of heat transfer.

### 2.1. High Thermal Conductivity Fibers

Heat conduction serves as the main form of thermal transfer in solids. According to Fourier’s law, aside from the temperature difference at the ends of the medium, the thermal conductivity of the material itself plays the most crucial role in determining the heat transfer rate in solids. Therefore, the key to designing functional fibers for thermal conduction lies in controlling heat transfer by engineering the material’s thermal conductivity. In solids, heat is mainly transmitted via electrons or phonon vibrations. The Wiedemann–Franz law is expressed as:

(1) $$k_{e}=\sigma LT$$

where *k_e_* is thermal conductivity, *T* is temperature, and *L* is the Lorenz number (a constant in metals), indicates that for conductive materials, the electron contribution to thermal conductivity is directly related to electrical conductivity. In crystalline dielectric materials, thermal conductivity is chiefly governed by phonon transport and scattering within the crystal lattice, which can be described by the Boltzmann transport equation (BTE) as proposed by Peierls [20]. The mechanism of thermal conduction in amorphous materials remains uncertain, although Slack [21] and Cahill et al. [22] have proposed theories on the minimum thermal conductivity of disordered solids based on the phonon gas model (PGM). On the microscale, the design of thermal materials and devices mainly depends on phonon transport and scattering. Many studies in nanoelectronics [23] have explored methods for adjusting thermal conductivity through coherent phonon transport [24], phononic crystals [25], and localized resonance [26]. Currently, depending on the application, the thermal properties of fibers are modified using compositional design, microstructural engineering, and assembling diverse materials to meet functional requirements such as thermal insulation or heat dissipation.

In the case of solids, heat is generated by the vibrations of microscopic particles, and heat conduction fundamentally involves the transfer of vibrational energy among these particles. The types and quantities of microscopic particles vary considerably across different materials. In metallic substances, the thermal motion of electrons is predominant, whereas in non-metallic substances, phonons serve as the primary agents [27]. To enhance the thermal conductivity of fibers, various strategies can be employed: utilizing materials with inherently high thermal conductivity, incorporating high-thermal-conductivity fillers into the fiber matrix, and optimizing the internal structure of fibers to establish efficient thermal conduction networks, among others [28,29,30,31].

**Figure 2:**
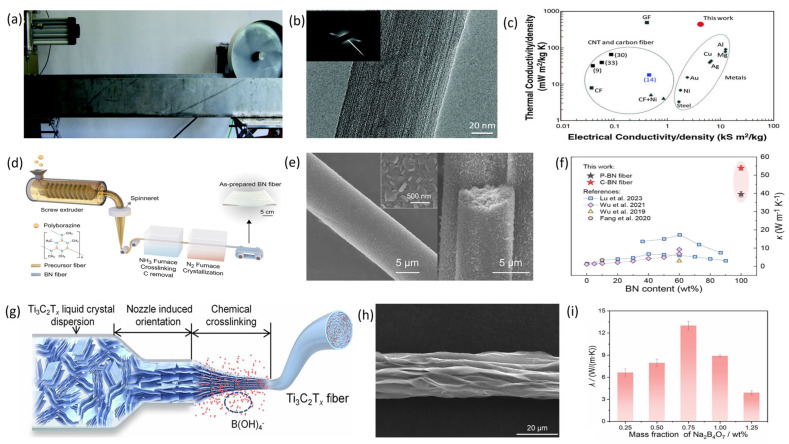
Fibers with inherently high thermal conductivity. (**a**) Schematic diagram of the carbon nanotube fiber spinning apparatus. (**b**) Transmission electron microscopy micrograph and electron diffraction pattern (inset) of a single fiber. (**c**) Relationship between specific electrical conductivity and specific thermal conductivity (Ashby chart). (**a**–**c**) reproduced with permission from [28]. Copyright (2013) AAAS. (**d**) Schematic illustration of the fabrication process for continuous high-quality boron nitride fibers. (**e**) Representative SEM image of a single BN fiber. (**f**) Comparison of the thermal conductivity (κ) of individual boron nitride (BN) fibers, including the pure BN fiber from this study and previously reported single composite fibers. (**d**–**f**) reproduced with permission from [41,43,44,45,46]. Copyright (2025) American Chemical Society (**g**) Schematic diagram of the Ti_3_C_2_T_x_ fiber fabrication process. (**h**) Scanning electron microscopy image of Ti_3_C_2_T_x_ fibers. (**i**) Thermal conductivity (λ) of Ti_3_C_2_T_x_ fibers. (**g**–**i**) reproduced with permission from [42]. distributed under the terms of the Creative Commons Attribution License (CC BY 4.0).

The initial category consists of fiber materials characterized by inherently high thermal conductivity coefficients. Based on their material properties, these can be subdivided into metallic and non-metallic materials. Metals include silver (429 W·m^−1^·K^−1^), copper (398 W·m^−1^·K^−1^ gold (315 W·m^−1^·K^−1^), W·m^−1^·K^−1^aluminum (247 W·m^−1^·K^−1^), and tungsten (173 W·m^−1^·K^−1^). Non-metallic materials are further classified into carbon-based fibers and ceramic fibers. Carbon-based fibers were first developed in 1960 by the Japanese scientist Akio Shindo, utilizing polyacrylonitrile as the base material [32]. Subsequently, in the early 1960s, pitch-based carbon fibers were produced through high-temperature carbonization processes. Compared to polyacrylonitrile-based fibers, these exhibit superior stability, improved mechanical properties, and higher thermal conductivity [33]. The K1100 carbon fiber, developed by Cytec Industries in the United States, attains a maximum thermal conductivity of 1100 W·m^−1^·K^−1^. Pitch-based carbon fibers [34] are categorized into general-purpose fibers and mesophase pitch-based fibers, based on performance. Mesophase pitch-based carbon fibers are derived from mesophase pitch via melt-spinning. Due to the high molecular weight, increased graphitization, and crystallinity of mesophase pitch, the resulting fibers possess a well-developed graphitic structure with axially aligned graphene units. These microstructural features significantly contribute to their exceptional thermal conductivity and mechanical performance [35]. An additional promising carbon-based fiber is the carbon nanotube (CNT) fiber. CNTs are tubular, one-dimensional nanomaterials that demonstrate remarkable thermal conductivity, reaching up to 3500 W·m^−1^·K^−1^, owing to their structure, primarily composed of sp^2^-hybridized carbon atoms interconnected by *σ* and π bonds. CNT fibers are macroscopic fibers assembled from parallel-aligned carbon nanotubes serving as fundamental building blocks. Common fabrication methodologies include floating catalyst chemical vapor deposition (FCCVD), array spinning, and wet spinning. In 2000, French scientists Vigolo et al. first synthesized CNT fibers using wet spinning technology [36]. Subsequently, in 2002, Professor Wu Dehai at Tsinghua University and Professor Ajayan at Rensselaer Polytechnic Institute collaborated to produce CNT bundles measuring up to 20 cm in length and with diameters ranging from approximately 300 to 500 μm via FCCVD [37]. In 2013, Professor Pasquali at Rice University in the US used chlorosulfonic acid to disperse carbon nanotubes and employed liquid crystal spinning to produce carbon nanotube fibers (see Figure 2a), achieving a thermal conductivity of up to 635 W·m^−1^·K^−1^ (see Figure 2b,c) [28]. The final type of carbon-based fiber is graphene fiber. Graphene is a two-dimensional nanomaterial consisting of a single atomic layer, with single-layer graphene reaching an extremely high thermal conductivity of up to 5300 W·m^−1^·K^−1^ [38]. This high thermal conductivity is mainly due to phonon scattering. Graphene’s excellent thermal transport properties are primarily driven by internal phonon heat transfer, facilitated by strong in-plane chemical bonds that enable phonon conduction, resulting in high in-plane thermal conductivity [39]. In 2011, Gao Chao’s research team at Zhejiang University first reported producing graphene fibers through wet-spinning [40]. Liao et al. [41] successfully produced pure boron nitride fibers with high thermal conductivity using a polymer-derived ceramics method, as shown in Figure 2d,e. The thermal conductivity of a single pure boron nitride fiber, measured with the big-MEMS method, reached 54 W·m^−1^·K^−1^ (see Figure 2f). When these fibers were used as thermal enhancement materials, polymer composites reinforced by a “stacking and cutting” technique achieved a thermal conductivity of 24.4 W·m^−1^·K^−1^ at 65 wt% loading. Zhou et al. [42] developed a new approach to making high-performance Ti_3_C_2_T_x_ fibers. By adding small amounts of borate during the wet-spinning of Ti_3_C_2_T_x_ fibers (see Figure 2g), the fiber microstructure shown in Figure 2h was obtained. Borate forms strong interlayer cross-links with hydroxyl groups on Ti_3_C_2_T_x_’s surface, strengthening interlayer interactions, improving nanosheet alignment, reducing internal pores, and enhancing fiber properties, including a thermal conductivity of up to 13 W·m^−1^·K^−1^ (see Figure 2i). This strategy offers new insights into the functional development of nanofibers. Ceramic materials with high thermal conductivity encompass diamond, boron nitride, silicon carbide, and aluminum nitride, among others. These ceramics exhibit excellent insulating properties, corrosion resistance, high toughness, and in theory, high single-crystal thermal conductivity, rendering them promising for application in fields such as aerospace engines, high-temperature bearings, corrosion-resistant components, and electronic devices. However, due to the stringent requirements inherent in ceramic fabrication processes, materials produced through conventional methods often contain numerous lattice defects and residual impurities. Additionally, limitations in fabrication conditions can lead to significant variations in grain size, morphology, and the content and distribution of secondary phases at grain boundaries, resulting in a notable discrepancy between the actual thermal conductivity of the ceramics and their theoretical single-crystal thermal conductivity. Therefore, ceramic materials are frequently utilized as fibrous fillers, such as flakes or particles, within composite fibers to enhance their thermal conductivity.

**Figure 3:**
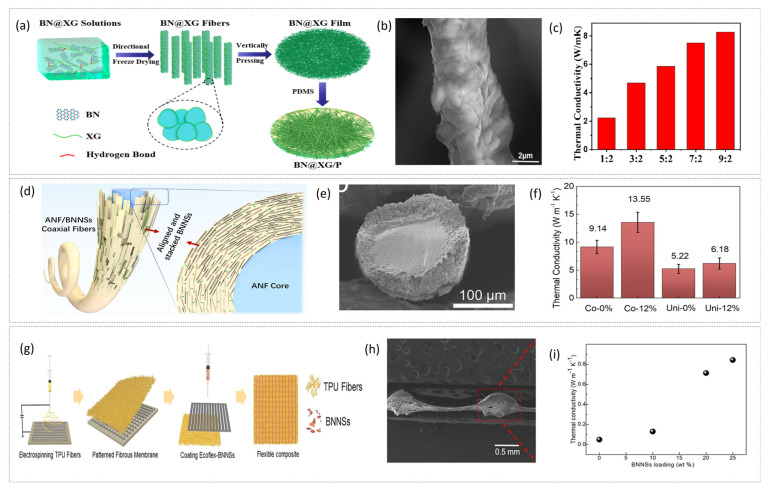
Thermally conductive functional fibers incorporating high thermal conductivity fillers. (**a**) Schematic diagram of the synthesis process of BN@XG/P. (**b**) Scanning electron microscopy (SEM) image of freeze-dried BN@XG fibers. (**c**) In-plane thermal conductivity of the prepared BN@XG/P film. (**a**–**c**) reproduced with permission from [51]. Copyright (2024) (Wiley, Hoboken, NJ, U.S.) (**d**) Schematic diagram of the surface and cross-sectional structure of ANF/BNNSs coaxial fibers. (**e**) SEM image of the cross-section of coaxial fibers. (**f**) Comparative analysis of the thermal conductivity of coaxial and uniaxial fibers with 50 wt% BNNSs content. Samples Co-0%/Uni-0% and Co-12%/Uni-12% represent the comparison of coaxial/uniaxial fibers subjected to 0% and 12% tensile strain during hot stretching (κ), including pure BN fibers in this study and previously reported single composite fibers. (**d**–**f**) reproduced with permission from [43]. distributed under the terms of the Creative Commons Attribution License (CC BY 4.0). (**g**) Schematic diagram of the process for preparing composite fibers by coating TPU surfaces with BNNSs. (**h**) SEM image of the cross-section of a fiber mesh coated with an Ecoflex-BNNSs composite layer. (**i**) Thermal conductivity of samples with different BNNSs contents. (**g**–**i**) reproduced with permission from [52]. distributed under the terms of the Creative Commons Attribution License (CC BY 4.0).

The second strategy for constructing high-thermal-conductivity fibers involves adding materials with high natural thermal conductivity as fillers into traditional fibers. This type of fiber represents a typical additive-modified fiber, which enhances thermal conductivity by uniformly dispersing thermally conductive fillers within a single polymer matrix. Unlike bicomponent fibers, these fibers do not form a dual-phase structure but rather optimize performance through filler distribution and interface engineering. Common high-thermal-conductivity fillers include carbon nanotubes (CNTs) [47], graphene [48], boron nitride (BN) [49], and transition metal carbides/nitrides (MXenes) [50], among others. Ye et al. [51] developed a thermally conductive fiber membrane made from interconnected boron nitride nanosheet-based self-assembled structures for high-performance thermal interface applications. As shown in Figure 3a, a BN solution at a specific concentration was evenly mixed with xanthan gum (XG), then subjected to directional freeze-casting and freeze-drying to produce BN@XG fibers (Figure 3b). These fibers were physically compressed and immersed in polydimethylsiloxane (PDMS or P) to create the thermal interface film BN@XG/P. The results indicated that both in-plane and through-plane thermal conductivities increased as boron nitride content rose (Figure 3c). The maximum in-plane thermal conductivity reached 8.26 W·m^−1^·K^−1^, a 50-fold increase over pristine XG film. This demonstrates that boron nitride bi-component fibers construct an effective thermally conductive network within the film, greatly enhancing its thermal performance. Lu et al. [43] produced hexagonal boron nitride nanosheet/polymer bi-component fibers via coaxial wet-spinning, achieving a high degree of nanosheet alignment within the fibers. The thermal stretching process in coaxial wet-spinning created compressive forces between the core and shell, promoting axial alignment of the nanosheets (Figure 3d). As shown in Figure 3e, the nanosheets densely stacked around the circular core showed an orientation factor of 0.81, as determined by wide-angle X-ray scattering. In comparison, uniaxial-spun fibers displayed more randomly oriented nanosheets at the fiber center, with an orientation factor near 0.65. The thermal conductivity of coaxial-spun fibers increased steadily with boron nitride nanosheet content, reaching 17.2 W·m^−1^·K^−1^ at 70 wt% loading (Figure 3f). Under the same conditions, uniaxial-spun fibers only achieved 6.74 W·m^−1^·K^−1^, about one-third of the coaxial-spun fibers. These findings highlight that well-aligned hexagonal boron nitride nanosheets can significantly improve the thermal conductivity of composite fibers. Sun et al. [52] created a composite fiber with advanced thermal management and high permeability. Their process, shown in Figure 3g, involved fabricating patterned electrospun thermoplastic polyurethane (TPU) fibers using a metal grid collector, then coating the patterned TPU with Ecoflex-BNNSs nanostructures (Figure 3h). Ecoflex offered excellent elasticity and amorphous characteristics, ensuring flexibility, while boron nitride nanosheets enhanced thermal conductivity. Thermal bridge measurements showed that a composite with 25 wt% BNNSs had a thermal conductivity of 0.844 W·m^−1^·K^−1^ (Figure 3i), representing a 4442% increase over pure patterned TPU fibers (0.019 W·m^−1^·K^−1^). This confirms that boron nitride nanosheets effectively boost thermal performance. When integrated into flexible devices, the composite maintained significantly lower stable operating temperatures compared to pure Ecoflex encapsulation. Over 2000 testing cycles, temperature fluctuations on the surface remained below 0.5 °C.

**Figure 4:**
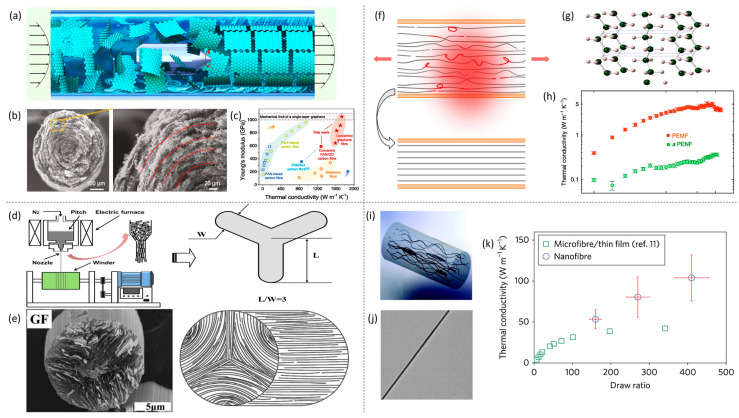
Regulating the Internal Structure of High Thermal Conductivity Fibers. (**a**) illustrates the evolution of the ordered layered structure within the graphene fiber spinning tube under the influence of a rotational shear composite multi-shear flow field. (**b**) Cross-sectional SEM image of graphene oxide aerogel fiber under a multi-shear flow field, The red dashed line illustrates the concentric circular texture characteristics of graphene oxide sheets in the cross-section of graphene fibers. (**c**) Ashby plot of Young’s modulus versus thermal conductivity, including the prepared concentric graphene fibers, previously reported graphene fibers, and polyacrylonitrile-based/pitch-based carbon fibers. (**a**–**c**) reproduced with permission from [53]. distributed under the terms of the Creative Commons Attribution License (CC BY 4.0). (**d**) Schematic diagram of a spinneret with a Y-shaped spinning orifice for producing high thermal conductivity mesophase pitch-based graphite fibers. (**e**) Cross-sectional SEM image of pitch-based graphene and a schematic diagram of its fiber structure. (**d**,**e**) reproduced with permission from [54]. distributed under the terms of the Creative Commons Attribution License (CC BY 4.0). (**f**) Idealized schematic of producing PENF from polyethylene microfiber (PEMF) using localized stretching with a micro-heater, where the arrow indicates the direction of mechanical stretching. (**g**) Orthorhombic crystal structure of polyethylene. Scale bar is 500 nm. (**f**,**g**) reproduced with permission from [58]. distributed under the terms of the Creative Commons Attribution License (CC BY 4.0). (**h**) dk(T) relationship curves for PEMF and amorphous (a)PENF. Red dots represent data for PEMF before localized stretching, green dots represent data for c-PENF material transformed into an amorphous state due to focused ion beam (FIB) irradiation. (**i**) Schematic diagram of the structure of polyethylene microfiber after mechanical stretching. (**j**) Transmission electron microscopy image of ultra-drawn polyethylene nanofiber. (**k**) Plot of the thermal conductivity of ultra-drawn polyethylene fiber samples versus their corresponding draw ratio. (**h**,**i**) reproduced with permission from [56]. Copyright (2010) Springer Nature.

The third strategy involves enhancing the thermal conductivity of fibers by establishing efficient and unobstructed heat conduction pathways through the regulation of internal component structures within the fibers. Gao et al. [53] successfully created graphene fibers with extremely high thermal conductivity using a multi-shear flow-assisted wet-spinning (MSW) method. During this process, they innovatively employed a radial rotational flow field, enabling precise control over the orderly assembly of graphene sheets both longitudinally and transversely by modulating the shear flow. This approach produced graphene fibers with a concentric texture of graphene oxide sheets in cross-section, as shown in Figure 4a,b. Optimizing the alignment of these sheets resulted in a denser, more crystalline graphitic structure, which significantly improved the fibers’ properties. The thermal conductivity of the fibers reached 1590 W·m^−1^·K^−1^, about 16% higher than conventional graphene fibers (Figure 4c). The researchers also demonstrated that this concentric structure could be applied to make highly thermally conductive polyacrylonitrile (PAN)-based carbon fibers. Cao et al. [54] described a simple method to prepare high-thermal-conductivity, circular cross-section mesophase pitch-based graphite fibers (MPGFs). As illustrated in Figure 4d, they first produced triangular cross-section precursor pitch fibers via melt-spinning through Y-shaped spinnerets. These fibers were then subjected to graphitization, resulting in MPGFs with circular cross-sections. The strong shear effects from the Y-shaped orifices caused high alignment of polycyclic aromatic hydrocarbons along the fiber axis. Characterization showed that the fibers possessed large crystal sizes and an exceptionally high orientation degree of 95.9%. As shown in Figure 4e, the crystallite arrangement was clearly visible, with the graphite cross-section appearing mostly circular and the crystallites exhibiting a distinct Y-shaped radial alignment. These graphite fibers achieved a thermal conductivity of up to 1070 W·m^−1^·K^−1^. For polymer fibers, enhancing thermal conductivity generally involves increasing molecular chain alignment or reducing amorphous regions. These improvements can be achieved by processing polymers into films or fibers [55]. Some commercially available polyethylene (PE) fibers, produced via spinning-induced alignment, exhibit thermal conductivities between 30 and 40 W·m^−1^·K^−1^ at room temperature. Shen et al. [56] demonstrated that ultrahigh-molecular-weight polyethylene (UHMWPE) nanofibers with a thermal conductivity of 104 W·m^−1^·K^−1^ could be made through mechanical stretching of polymer chains. As shown in Figure 4h,i, during initial stretching, small crystalline blocks detached from lamellae and transformed into microfibrils aligned with the stretch direction. At low stretching ratios, these crystalline blocks remained interconnected by entangled tie molecules, originating from partially unfolded chains. Microfibrils were also linked by bridging molecules. With increased stretching, the microfibrils further disentangled and aligned under shear forces, reducing their volume fraction and increasing the number of fully unfolded tie molecules, which led to larger crystal sizes along the stretch. Higher stretching ratios increased the volume fraction of chain-oriented crystalline regions, thus enhancing thermal conductivity. As shown in Figure 4f, Shrestha et al. [57] produced highly aligned, crystalline PE nanofibers through localized heating and stretching. Their thermal conductivity increased with temperature, reaching a maximum of 90 W·m^−1^·K^−1^ at 130 K (Figure 4g). Electrospinning is another effective technique for producing aligned polymer nanofibers. Ma et al. [58] studied the relationship between molecular orientation and thermal conductivity in electrospun polyethylene nanofibers under different electric field conditions. During electrospinning, the applied voltage caused strong stretching of the PE chains, resulting in high molecular orientation and crystallinity. Tests confirmed that higher voltages increased chain alignment and crystallinity, which improved phonon transport within the fibers and raised thermal conductivity. At 45 kV, the highest measured thermal conductivity reached 9.3 W·m^−1^·K^−1^, well above the 0.4 W/m·K typical of bulk PE. Zhang et al. [59] developed a high-performance thermally conductive composite, PP@G, using polypropylene (PP) from discarded face masks and graphene nanoplatelets (GNPs) via an electrostatic self-assembly strategy. This approach enables the formation of continuous and efficient thermal conduction pathways along PP fiber surfaces by aligning GNPs, thereby enhancing the composite fiber’s thermal conductivity to 87 W·m^−1^·K^−1^ This method demonstrates notable advantages in reducing material costs and constructing environmentally friendly materials.

In summary, as a vital material in the field of thermal management, high thermal conductivity fibers have seen significant research progress in recent years; Table 1 summarizes the performance characteristics of various high thermal conductivity fibers. Researchers primarily use three design and fabrication strategies to improve the thermal conductivity of fibers. The first strategy involves employing fibers with naturally high thermal conductivity, such as metallic, ceramic, and carbon-based materials, exemplified by pitch-based carbon fibers. The second strategy focuses on incorporating thermally conductive fillers (e.g., carbon nanotubes, boron nitride nanosheets) with high thermal conductivity into conventional fibers through composite spinning techniques like coaxial spinning and electrospinning to create composite fibers. The third strategy aims to establish efficient thermal conduction pathways by applying external forces (e.g., electric fields, mechanical forces) during the artificial spinning process to control the organized distribution and orientation of fibers or internal fillers, thereby enhancing thermal conductivity. Although these approaches can effectively boost the thermal conductivity of fibers, several challenges still hinder the design, manufacturing, and application of high-performance, thermally conductive fibers. Firstly, the compatibility between fillers and the matrix within composite fibers remains a major concern. Additionally, controlling filler loading and distribution within fibers lacks effective methodologies. Secondly, many advanced fabrication techniques, such as ice-templating and chemical vapor deposition, involve complex procedures and high costs, limiting large-scale industrial implementation. Furthermore, while thermal conductivity improves, high thermal conductivity fibers often struggle to balance other properties, such as mechanical strength, chemical stability, and environmental resilience. Additionally, the micro/nano-scale assessment of thermal conductivity remains inadequate due to a lack of mature commercial instruments. Although measurement methods like the T-type method, 3 ω method, and suspended microdevice are used for precise evaluation, challenges such as transferring micro-scale samples and interfacial thermal resistance between samples and measurement devices can negatively impact accuracy. Currently, as the application scenarios for thermally conductive functional fibers diversify, fiber design is evolving towards multifunctionality, high performance, and intelligence. In terms of material development, researchers continue to explore substances with superior thermal conductivity. Regarding fabrication processes, emphasis is increasingly placed on efficiency and cost-effectiveness, with green and sustainable techniques expected to become key areas in the future. Concerning functionality, the development of multifunctional fibers that incorporate electrical conductivity, radiation resistance, and high mechanical performance will be vital to meet the diverse requirements of various application environments.

### 2.2. Thermal Insulation Fibers

Unlike the goal of heat conduction, the purpose of thermal insulation is to slow down or stop heat transfer between two environments. It is widely accepted that materials with low density and porous structures, like foam materials, provide better thermal insulation. The effective thermal conductivity $\lambda_{eff}$ of insulating materials can be described by the following equation [60]:

(2) $$\lambda_{eff}=\lambda_{s,cond}+\lambda_{g,cond}+\lambda_{g,conv}+\lambda_{rad}$$

Here, $\lambda_{s,cond}$ represents the solid thermal conduction of the material, $\lambda_{g,cond}$ denotes the gas thermal conduction within the material, $\lambda_{g,conv}$ signifies the gas convective heat transfer inside the material, and $\lambda_{rad}$ stands for radiative heat transfer. When the material exhibits high porosity and the internal pore diameter is less than 1 mm, the movement of air within the pores is restricted to short distances, thereby effectively suppressing gas convective heat transfer. Under these conditions, radiative heat transfer can also be considered negligible. Consequently, the thermal conductivity of the material is predominantly influenced by internal solid thermal conduction and the gas thermal conduction within the voids. Gas thermal conduction is dictated by the pore diameter and the mean free path of gas molecules within the pores. Typically, when the pore diameter is smaller than the mean free path of air molecules (70 nm), heat transfer between these molecules is significantly hindered. According to the Knudsen effect [61], reducing the pore size of porous materials can enhance their thermal insulation properties. Notario et al. demonstrated that decreasing the pore diameter from 100 nm to 10 nm results in a substantial reduction in the thermal conductivity of polymer foams, from 17 W/m·K to 7 W/m·K [62]. As most thermal insulation materials are non-metallic, their internal solid thermal conduction mainly depends on phonon transport [63], and the thermal conductivity can be expressed as:

(3) $$\lambda_{s,cond}=c_{ph}\cdot l_{ph}\cdot v_{ph}/3$$

Here, $c_{ph}$ represents the specific heat capacity, $l_{ph}$ denotes the mean free path, and $v_{ph}$ is the velocity. According to the aforementioned equation, the suppression of solid heat conduction can be achieved by increasing the phonon scattering interfaces within the material. Enhancing the porosity of the material and reducing the size of internal pores effectively facilitates the construction of gas–solid interfaces, thereby elevating the interfacial thermal resistance of the solid and enhancing the material’s thermal insulation performance [64]. Shrestha et al. developed a comprehensive model capable of describing various forms of heat transfer behavior within thermal insulation materials [65], which can, to some extent, inform the design of high-performance thermal insulation materials. Based on theoretical analysis, foams and aerogels with abundant micropores and complex interfaces are considered promising materials with superior thermal insulation properties.

Aerogel fibers distinguish themselves within the domain of thermal insulation materials owing to their ultra-high porosity, extensive specific surface area, and exceedingly low thermal conductivity [66,67]. The thermal efficacy of aerogel fibers is contingent upon factors such as fiber diameter, internal architecture, and the thickness of the final product. These fibers are produced utilizing nanoscale building blocks through various methodologies, including confined spinning, freeze spinning, wet spinning, and liquid crystal spinning, thereby yielding functional fibers with a spectrum of structural compositions. Depending on the type of nanoscale building blocks employed, aerogel fibers can be classified into carbon-based, ceramic matrix, and polymer-based variants. The dimensions of these nanoscale building blocks also exhibit variation, encompassing zero-dimensional nanoparticles (e.g., silica, TiO_2_) [68,69], one-dimensional nanofibers (e.g., silver nanowires, cellulose nanofibers) [70], and two-dimensional nanosheets (e.g., graphene nanoplatelets) [71].

**Figure 5:**
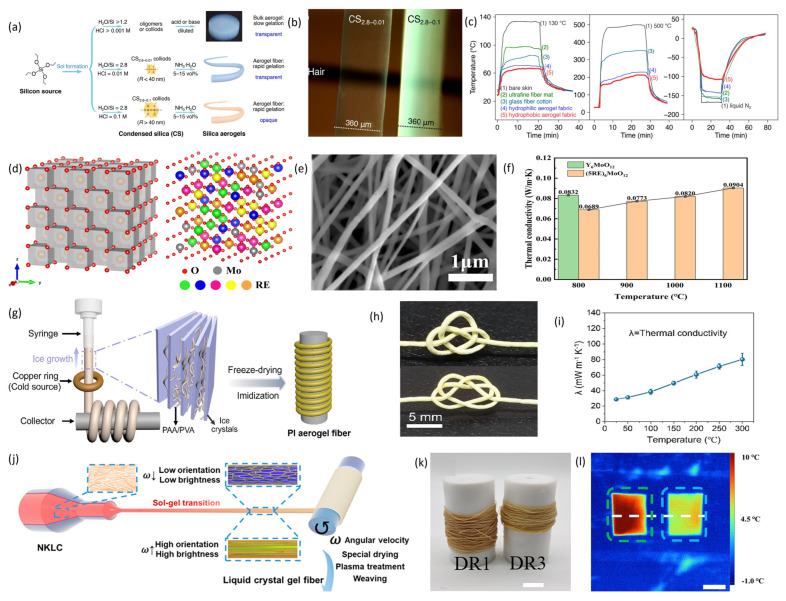
Insulating fibers of different materials. (**a**) Schematic diagram of the preparation of silica aerogel fibers by reactive spinning. (**b**) Optical micrographs of CS2.8–0.01 and CS2.8–0.1 samples on the surface of hair. (**c**) Comparison of the thermal insulation performance of silica aerogel fibers with glass fiber wool and microfiber felt. (**a**–**c**) reproduced with permission from [73]. Copyright (2020) American Chemical Society. (**d**) Unit cell model of the cubic defect fluorite structure (5RE)_6_MoO_12_. (**e**) SEM image of (5RE)_6_MoO_12_ nanofibers treated at 800 °C. (**f**) Room-temperature thermal conductivity of (5RE)_6_MoO_12_ nanofiber-based ceramics and Y_6_MoO_12_ nanofiber-based ceramics after sintering at different temperatures. (**d**–**f**) reproduced with permission from [74]. Copyright (2025) Elsevier. (**g**) Schematic diagram of the preparation process for polyimide aerogel fibers. (**h**) Photographic image of a polyimide aerogel fiber knot. (**i**) Thermal conductivity (λ) of polyimide aerogel fabric at different ambient temperatures. (**g**–**i**) reproduced with permission from [75]. Copyright (2022) Springer Nature. (**j**) Schematic diagram of the preparation process for nanoscale Kevlar liquid crystal (NKLC) aerogel fibers. (**k**) Micrographs of DR1 gel fiber (left) and DR3 gel fiber (right). Scale bar: 1 cm. (**l**) Thermal infrared images of DR3 aerogel fiber felt (left) and hollow cotton fiber felt (right) in equilibrium at 0 °C. Scale bar: 1 cm. (**j**–**l**) reproduced with permission from [76]. distributed under the terms of the Creative Commons Attribution License (CC BY 4.0).

Silica, as an inorganic material, offers advantages such as fire resistance, high-temperature tolerance, and low cost. Silica aerogel fibers, derived from silica aerogels, have been employed in the field of thermal protection for several decades. Pico et al. [68] demonstrated the feasibility of producing silica aerogel fibers via a sol–gel process, resulting in fibers with a pore structure comparable to that of bulk silica aerogels. Subsequently, Meng et al. [72] developed continuous silica aerogel fibers with hollow structures using a wet-spinning process. Zhang et al. [73] achieved rapid gelation during fiber spinning by carefully controlling the molar ratio of H_2_O:TEOS and the HCl concentration in the solution, resulting in highly transparent silica aerogel fibers (Figure 5a). These fibers showed a more uniform silica particle distribution and higher specific surface area. Their transparency reached 92% (Figure 5b), with porosity of 94%, exceeding the 91% of opaque fibers. The estimated thermal conductivity ranged from 0.018 to 0.023 W·m^−1^·K^−1^, indicating strong potential for thermal management (Figure 5c). Deng et al. [74] introduced a novel ceramic nanofiber with low thermal conductivity by synthesizing a rare-earth-based high-entropy molybdate ceramic fiber, (Y_0.2_La_0.2_Er_0.2_Ho_0.2_Tm_0.2_)_6_MoO_12_, through electrospinning (Figure 5e). High-entropy ceramics (HECs) possess unique properties due to high-entropy and lattice distortion effects (Figure 5d). The (5RE)_6_MoO_12_ nanofibers combine HEC benefits with a fibrous structure, showing high porosity (83.45%) and ultra-low thermal conductivity (0.0689 W·m^−1^·K^−1^ at 25 °C) (Figure 5f). Their thermal stability makes them promising for insulation in extreme environments like aerospace. Compared to inorganic ceramic materials, polymeric aerogel fibers offer better tensile strength, flexibility, and large-scale processing advantages. Organic aerogel fibers have also advanced significantly recently. Liu et al. [75] created lightweight polyimide (PI) aerogel fibers with controllable pore structures via freeze-spinning (Figure 5h). As shown in Figure 5g, using polyvinyl alcohol (PVA) as an ice-nucleating agent, the interactions between PAA and PVA, as well as PVA and water, effectively reduced pore size. SEM images showed that PI aerogel fibers with 20% PVA had an average pore size of 11.5 ± 4.5 μm, significantly smaller than pure PI fibers. These fibers had high porosity (95.6%) and considerable mechanical strength. Their thermal conductivity was just 28.7 ± 2.0 W·m^−1^·K^−1^ at 25 °C, increasing slightly at 300 °C (Figure 5i). Zhang et al. [76] designed nanoscale Kevlar aerogel fibers with tunable internal structures. Using Kevlar nanofibers, they employed liquid crystal wet-spinning, dynamic sol–gel transition, freeze-drying, and cold plasma treatment to produce fibers with controlled, ordered microstructures (Figure 5j). By varying Kevlar nanofiber concentration and stretching ratio, they prepared NKLC gel fibers with different orientations (Figure 5k). These NKLC fibers demonstrated excellent thermal insulation (0.037 W·m^−1^·K^−1^). Figure 5l shows thermal infrared images of the DR3 aerogel fiber mat (left) and hollow cotton fiber mat (right) at 0 °C.

**Figure 6:**
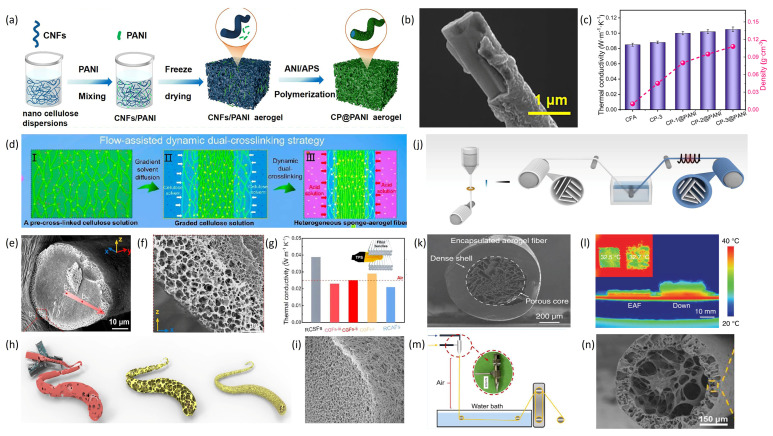
Aerogel Fibers with Reduced Thermal Conductivity through Structural Design. (**a**) Schematic illustration of the preparation method for CP-3@PANI aerogel. (**b**) The CP-3@PANI aerogel shows a more distinct polyaniline shell structure on the surface of the carbon nanofibers. (**c**) Thermal conductivity and density data of various aerogels, indicating their excellent thermal insulation properties. (**a**–**c**) reproduced with permission from [77]. distributed under the terms of the Creative Commons Attribution License (CC BY 4.0). (**d**) Strategy schematic for preparing CGFs with gradient pore structures (porous shell and dense fiber core) via a microfluidic spinning system. (**e**,**f**) Cross-sectional morphology of CGFs perpendicular to the fiber axis. (**g**) Measurement of the thermal conductivity of regenerated cellulose materials using a Transient Plane Source (TPS) device. (**d**–**g**) reproduced with permission from [78]. Copyright (2022) American Chemical Society. (**h**) Schematic diagram of the nanostructural changes during the preparation of GAFs, including ANF dispersion in the microfluidic system, sol (red)-gel (yellow) phase transition in the coagulation bath, and ethanol solvent exchange process. (**i**) Typical SEM image of gradient nanostructured aerogel fibers. (**h**,**i**) reproduced with permission from [79]. distributed under the terms of the Creative Commons Attribution License (CC BY 4.0) (**j**) Preparation of EAFs via freeze-spinning combined with a two-step coating-drying method. (**k**) Scanning electron microscopy image of an EAF. (**l**) Comparison under 40 °C experimental conditions: optical and infrared images of approximately 1 mm thick encapsulated aerogel textile material and approximately 5 mm thick down, both exhibiting equivalent thermal insulation performance. (**j**–**l**) reproduced with permission from [82]. Copyright (2023) AAAS. (**m**) Schematic of the stepwise coagulation coaxial wet-spinning process and the post-treatment procedure for preparing CA/PAA@CNF aerogel fibers. (**n**) Longitudinal and cross-sectional SEM images of CA/PAA@CNF aerogel fibers. (**m**,**n**) reproduced with permission from [80]. Copyright (2022) American Chemical Society.

In addition to modifying the types and properties of the nanoscale building blocks of aerogel fibers, various spinning strategies have been employed in recent years to design and optimize the internal structure of aerogel fibers to regulate their thermal conductivity. Chen et al. [77] described a CNF/PANI composite aerogel fiber with a core–shell structure. As shown in Figure 6a, polyaniline (PANI) was synthesized directly on the surface of electrospun carbon nanofibers to create composite aerogel fibers with PANI-coated CNFs (see Figure 6b). PANI formed effective links between CNFs, resulting in a stable three-dimensional network. The hybrid aerogel (CP-3@PANI) demonstrated a thermal conductivity of 0.104 W·m^−1^·K^−1^ and excellent flame-retardant properties (Figure 6c). Ye et al. [78] introduced a flow-assisted dual-crosslinking method to produce a fully cellulose hierarchical sponge aerogel fiber (CGF) using regenerated cellulose as the base material (Figure 6d). A microfluidic chip was used to control the flow of the cellulose core during spinning, enabling continuous production of CGFs with heterogeneous structures. As shown in Figure 6e,f, the CGF features a porous sponge outer layer and a dense aerogel core. These fibers have an adjustable sponge layer thickness and very low thermal conductivity (0.023 W·m^−1^·K^−1^). This method offers a new way to create gradient biomass fibers and holds promise for thermal insulation textiles. Fu et al. [79] developed a gradient all-nanostructured aramid aerogel fiber (GAF). Using microfluidic spinning, they achieved a gradient structure with a loose exterior and a dense interior (Figure 6h,i). After supercritical drying, the aerogel fibers exhibited a distinct gradient structure, with a porous core (~500 nm pores) and a shell (~160 nm pores). Their nanostructure, with porosity over 90%, resulted in high interfacial thermal resistance and excellent insulation, with a radial thermal conductivity of 0.0228 W·m^−1^·K^−1^—a 30–67% reduction compared to wet-spun aerogel fibers. Zhao et al. [80] developed a continuous process for making aerogel fibers with a polymer-encapsulated structure. Using stepwise coagulation, coaxial wet spinning, and freeze-drying, they produced CA/PAA-coated CNF aerogel fibers (Figure 6m), with the outer shell composed of cellulose nanofibers and a core of cellulose acetate/polyacrylic acid (CA/PAA) (Figure 6n). The inner core reached an ultrahigh porosity of 99.34%, and the hierarchical pores contributed to a low thermal conductivity of 0.054 W·m^−1^·K^−1^n addition, there exists a unique category of natural fibers in nature that hold significant applications in thermal insulation, namely animal fur-based fibers represented by wool. Wool-based textile materials are widely recognized for their exceptional thermal insulation properties, a characteristic attributed to their inherent crimped structure and the substantial volume of trapped static air within the fiber assemblies. As one of the most historically utilized and extensively applied natural insulating materials, wool serves as a crucial benchmark for understanding how microstructural features such as fiber curvature, hierarchical porous architecture, and low thermal conductivity influence thermal insulation performance [81]. Notably, the polar bear, as a representative species of frigid regions, possesses fur with outstanding thermal insulation properties, offering valuable insights for the design of advanced insulating fibers. Wu et al. [82] modeled the core–shell structure of natural polar bear hair to develop an encapsulated aerogel fiber (EAF). This fiber was produced via a two-step process (Figure 6j): first, freeze-spinning created the aerogel fiber with a controllable pore structure; second, it was coated with thermoplastic polyurethane (TPU) and freeze-dried to form a biomimetic core–shell structure (see cross-section in Figure 6k). The inner porosity exceeded 90%, while the TPU outer layer provided high strength and stretchability. Adjusting the internal structure improved the fiber’s mechanical properties and waterproofness, while maintaining excellent thermal insulation (Figure 6l), making it suitable for textile applications.

In summary, aerogel fibers, owing to their high porosity, low density, and intricate internal pore structure, offer promising opportunities for designing ultra-low thermal conductivity fibers. This makes them highly appealing for applications in thermal management under extreme conditions, such as personal thermal regulation, building insulation, and electronic device cooling. Table 2 summarizes the performance characteristics of various types of current aerogel fibers. Currently, the thermal insulation performance of aerogel fibers is mainly improved by increasing internal porosity, reducing pore size, and optimizing the fibers’ microstructure. Materials used for aerogel fibers can be broadly categorized into inorganic and organic types. Inorganic aerogel fibers, such as those made from silica and titanium dioxide, have low thermal conductivity and are cost-effective. However, they tend to be brittle, with limited mechanical strength and flexibility, making weaving difficult. Organic aerogel fibers, such as those from polyimide and cellulose, offer better flexibility, higher tensile strength, and enhanced thermal stability, which aids in weaving and device integration. Still, the intrinsic thermal conductivity of organic materials is relatively higher. Researchers are working on ways to enhance the mechanical and insulating properties of these fibers by refining spinning methods and developing more complex internal structures. Despite these advancements, challenges like complex manufacturing processes, high manufacturing costs, and the fibers’ sensitivity of thermal performance to humidity continue to limit the application and development of aerogel fibers.

### 2.3. Thermal Radiation-Regulating Fibers

Thermal radiation pertains to the phenomenon whereby any object possessing a temperature above absolute zero spontaneously and continuously absorbs and emits electromagnetic waves. Essentially, it constitutes a form of electromagnetic radiation. According to the Stefan–Boltzmann law [83]:

(4) $$E_{B}=\sigma_{0}T^{4}$$

where *σ* represents the Stefan–Boltzmann constant and *T* denotes the absolute temperature; it becomes evident that the radiation energy exhibits a high sensitivity to temperature changes. At lower temperatures, radiative heat transfer is virtually negligible, whereas at elevated temperatures, it becomes the principal mode of heat transfer. Furthermore, in accordance with Kirchhoff’s law of thermal radiation, the energy exchange attributable to thermal radiation can be expressed by the following equation [84]:

(5) $$Q_{\mathrm{rad}}=Q_{\alpha }+Q_{\rho }+Q_{\tau }$$

Herein, *α* represents the spectral absorption component, *ρ* denotes the spectral reflection component, and *τ* signifies the spectral transmission component. The proportion of each energy component relative to the total energy can be described by the absorptivity *α*, reflectivity *ρ*, and transmissivity *τ*, respectively [85]. Therefore, the radiative heat transfer of materials can be controlled through molecular design [86], surface modification [87], and microstructure design [88] to influence their infrared radiation properties. This allows for applications such as directional heating, thermal insulation, and cooling. Most objects near room temperature on Earth emit thermal radiation within the infrared wavelength range (0.76–1000 µm), which includes the near-infrared (0.75–2.5 μm), mid-infrared (2.5–25 μm), and far-infrared (25–1000 μm) regions. Since most organic and inorganic substances have fundamental absorption bands in the mid-infrared region, this area is the most widely studied and utilized. This section mainly reviews recent research on thermal radiation regulation fibers designed for the mid-infrared and near-infrared wavelengths.

**Figure 7:**
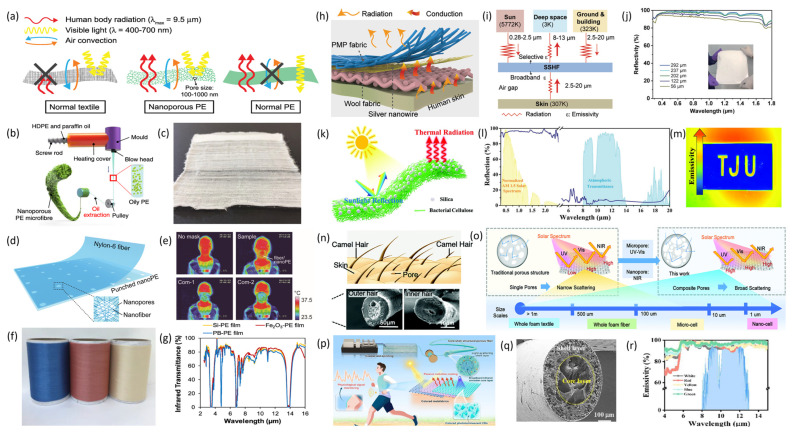
Radiative Cooling Functional Fibers. (**a**) Schematic comparison of the properties of nano-polyethylene, conventional polyethylene, and cotton fabric. Only nano-polyethylene simultaneously satisfies the three characteristics of infrared transmission, visible light blocking, and air convection. reproduced with permission from [86]. Copyright (2016) AAAS. (**b**) Schematic diagram of the manufacturing process for nano-PE microfibers. (**c**) Photograph of a knitted nano-polyethylene fabric. Scale bar: 3 cm. (**b**,**c**) reproduced with permission from [88]. Copyright (2018) Springer Nature. (**d**) Design schematic of an electrospun nylon-6 nanofiber mask on a needle-punched nano-PE substrate. (**e**) Thermal imaging analysis of bare skin, skin covered with the sample (fiber/nano-PE), and two types of commercial masks. (**d**,**e**) reproduced with permission from [92]. Copyright (2017) American Chemical Society. (**f**) Photograph of three rolls of colored polyethylene fibers produced by industrial extrusion. (**g**) Fourier-transform infrared transmittance of polyethylene composite materials embedded with inorganic pigments. (**f**,**g**) reproduced with permission from [93]. Copyright (2019) Cell Press. (**h**) Schematic of the multi-layer SSHF structure composed of PMP fabric, silver nanowires (AgNW), and wool fabric layers. (**i**) Schematic of the radiative heat transfer network of SSHF in an outdoor environment. (**j**) Ultraviolet-visible-near-infrared reflectance spectra of PMP fabrics with different thicknesses. Inset: photograph of the electrospun PMP fabric showing its white appearance. (**h**–**j**) reproduced with permission from [98]. Copyright (2024) AAAS. (**k**) Schematic illustrating the radiative cooling mechanism of nanostructured Bio-RC films. (**l**) Ultraviolet-visible-infrared reflectance spectrum of the nanostructured Bio-RC film. (**m**) Infrared thermal image of the nanostructured Bio-RC film with copper foil as a reference (blue area). (**k**–**m**) reproduced with permission from [99]. Copyright (2023) WILEY. (**n**) Schematic of the double-haired structure of camels. (**o**) Schematic comparison of the radiative cooling principles between MEPF and traditional porous fibers. (**n**,**o**) reproduced with permission from [101]. distributed under the terms of the Creative Commons Attribution License (CC BY 4.0). (**p**) Working principle schematic of CPRC meta-fabric woven from core–shell structured porous fibers produced by electrospinning. (**q**) Cross-sectional morphology of the core–shell structured porous fiber. (**r**) Mid-infrared emissivity of CPRC meta-fabrics in different colors. (**p**–**r**) reproduced with permission from [103]. Copyright (2025) American Chemical Society.

Radiative cooling, as an emerging passive cooling technology, shows great potential in building thermal protection and personal thermal management. Its main principle involves transferring radiant heat to the colder outer space through the atmospheric window (8–13 μm) [89,90]. Radiative cooling fibers can achieve cooling by two design strategies: increasing either the mid-infrared (MIR) transmittance or emissivity. Enhancing the MIR transmittance helps reduce the reflection of radiation from internal structures by the radiative surface, which lowers internal heat buildup and promotes cooling [91]. Since most organic and inorganic substances have fundamental absorption bands in the mid-infrared, materials that are naturally transparent to radiation are rare. Notably, polyolefins like polyethylene (PE), which only contain C–C and C–H bonds, are highly transparent in the mid-infrared range. To develop practical infrared-high-transmittance textiles, Hsu et al. [86] introduced nanoporous polyethylene (nanoPE) for personal cooling, featuring interconnected pores of 50–1000 nm that scatter sunlight and render the material opaque in visible light. These pores are significantly smaller than infrared wavelengths, permitting high infrared transmittance, as illustrated in Figure 7a. Peng et al. [88] successfully manufactured nanoPE fibers at scale via melt-spinning (refer to Figure 7b). These fibers exhibit high flexibility, resistance to abrasion, durability, and effective mid-infrared (MIR) transmission. The nanopores scatter visible light, rendering the fibers nearly 90% opaque visually, while transmitting over 70% of infrared radiation. The authors estimate that nanoPE fabrics (see Figure 7c) can reduce skin temperature by 2.3 °C compared to similarly thick cotton fabrics, thereby conserving over 20% of energy used for indoor cooling. Yang et al. [92] developed a nanofiber system utilizing electrospinning on micro-needle-perforated nanoPE (refer to Figure 7d), in which nanofibers efficiently capture particulate matter, and the PE substrate preserves high infrared (IR) transparency conducive to radiative cooling (see Figure 7e). This system is suitable for cooling face masks, with an IR transmittance of 92.1%, transmitting virtually all body IR radiation and providing effective radiative cooling at a low cost. Cai et al. [93] fabricated colored polyethylene fibers with high infrared transparency by blending inorganic pigment nanoparticles into the polyethylene (see Figure 7f). These colored fibers can be produced continuously with high strength through composite extrusion. Textiles made from these fibers exhibit an IR transmittance of 80% (see Figure 7g) and achieve nearly 2 °C of radiative cooling. Wang et al. [94] produced foamed thermoplastic polyurethane (TPU) fibers with anisotropic pores utilizing micro-extrusion foaming technology. The fibers possess hierarchical pores, which provide vibration damping within the mid-infrared range and reflect over 97% of near-infrared light, thereby enabling efficient passive radiative cooling. To attain high transparency, materials must be extremely thin, typically under 150 μm, which limits their applications. Enhancing mechanical properties and toughness within these constraints remains a challenge.

Enhancing the radiative cooling performance of materials primarily involves increasing the efficiency of heat dissipation from the local environment to the external surroundings. An alternative approach to radiative cooling is the utilization of materials characterized by high emissivity and high reflectivity. High reflectivity effectively reflects incoming radiative heat from the external environment, while enhancing outward emissivity facilitates the efficient radiation of internal heat through the atmospheric window, thereby achieving radiative cooling [95]. Typically, the refractive index of fibers is augmented by embedding high-refractive-index nanoparticles within the fibers or by constructing void structures inside polymer fibers to augment mid-infrared (MIR) scattering [96]. Improvement in emissivity is generally attained through material selection or the design of photonic structures [97]. Wu et al. [98] developed a mid-infrared (MIR) spectrally selective hierarchical fabric (SSHF) through molecular design. This fabric incorporates a poly(4-methyl-1-pentene) (PMP) nano-micro hybrid fiber layer, silver nanowires (AgNWs), and wool fabric (refer to Figure 7h). Electrospun PMP fibers, characterized by a broad size distribution, facilitate extensive scattering efficiency across the solar spectrum. The AgNW layer reflects MIR radiation efficiently, thereby preventing heat transfer to the human body, while wool’ s broadband emissivity absorbs radiative heat from the body (see Figure 7i). The SSHF demonstrates a high spectral selectivity ratio of 2.23 within the atmospheric transparency window (ATW) and an average ATW emissivity of 85% (see Figure 7j). It predominantly exhibits ATW-dominated emissivity on the outer surface, with broadband emissivity inward. Elevated emissivity in the ATW permits radiative heat dissipation into space, whereas high reflectivity in non-ATW regions curtails heat absorption from surrounding warmer environments. This spectrally selective design constitutes an innovative passive cooling solution for personal application. Shi et al. [99] produced a biosynthetic bacterial cellulose (BC)-based radiative cooling material, achieved by doping silica microspheres into a bacterial nanofibrous cellulose membrane, resulting in a freestanding nanostructured Bio-RC film featuring distinct cellulose and silica microsphere layers (see Figure 7k). This fiber-derived film exhibits a high solar reflectance of 95.3% and switchable solar transmittance levels of 4.7% (opaque) and 70% (transparent), as illustrated in Figure 7l. In its opaque configuration, it reduces daytime temperature by approximately 3.7 °C under solar irradiance of 647 W/m^2^. Wu et al. [100] developed a multilayer silk textile (MST) based on natural silk. Processed into silk fibroin solution and electrospun into silk nanotextiles, these fibers are subjected to hot-pressing at 60 °C and are sandwiched between commercial silk and ultrathin expanded polytetrafluoroethylene (e-PTFE), thereby forming the MST. The nanofabrics possess a solar reflectance of 93.4% and LWIR emissivity of 96.1%, with performance further enhanced to 96.5% and 97.1%, respectively. Inspired by the structure of camel hair (see Figure 7n), Wang et al. [101] engineered a physically foamed thermoplastic polyurethane (TPU) microporous elastic fiber (MEPF) and a biomimetic porous fabric (MEPFT-d). The foamed TPU fibers possess an internal microporous architecture, and the amalgamation of micropores and nanopores within the fibers renders the MEPF opaque white, thereby enhancing its solar reflectance to 98.7% and mid-infrared (MIR) emissivity to 97.2%. The cooling process of these fibers is depicted in Figure 7o. During daytime sunlight exposure, MEPFT-d attains a net cooling power of up to 3002 W/m^2^, with the cooling efficiency increasing as the ambient temperature elevates. Zhang et al. [102] developed a Janus-structured composite membrane via electrospinning and spray-coating, featuring radiative cooling enabled by an electrospun cellulose acetate (CA) fiber membrane. The IR emissivity, driven by molecular vibrations within CA, reaches 96%, while solar reflectance is facilitated by the membrane’s porous structure and fiber size heterogeneity. The average temperature of the CA fiber membrane was recorded to be 9.4 °C lower than that of cotton fabric. Liu et al. [103] designed a colored, photoluminescent passive radiative cooling (CPRC) coaxial porous fiber. Utilizing coaxial electrospinning, freeze-drying, and coating techniques, they fabricated core–shell fibers (see Figure 7p). The shell comprises cellulose acetate (CA) intercalated with h-BN nanosheets and a carbon dot (CD) coating, whereas the core incorporates single-walled carbon nanotubes (SWCNTs) (see Figure 7q). The nanoporous structure and h-BN in the shell enhance broadband scattering, thereby increasing solar reflectance. Concurrently, cellulose acetate and SWCNTs contribute to broadband MIR emissivity (see Figure 7r). The synergetic effect of the shell and core imparts the fibers with robust passive radiative cooling capabilities, achieving a maximum solar reflectance of 96.6% and MIR emissivity of 97.2%. Practical evaluations demonstrated a cooling effect of up to 5.1 °C.

**Figure 8:**
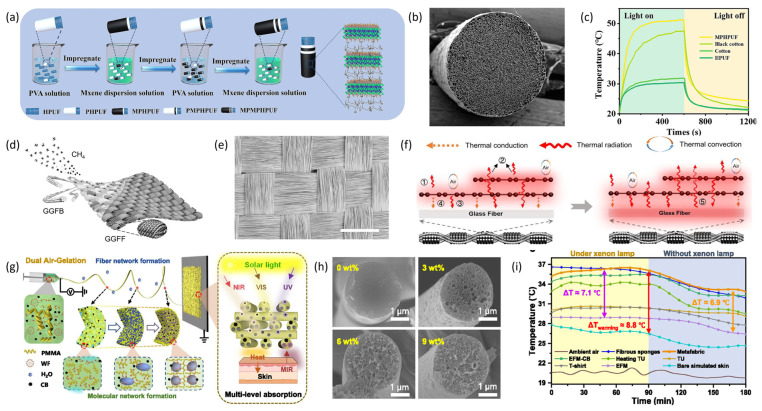
Radiative Heating Functional Fibers. (**a**) Schematic diagram of the MPHPUF fabrication process. (**b**) Cross-sectional SEM image of MPHPUF. (**c**) Real-time temperature variations in MPHPUF fabric, black cotton fabric, original white cotton fabric, and HPUF-60 fabric under an illumination intensity of 125 mW/cm^2^. (**a**–**c**) reproduced with permission from [104]. Copyright (2024) Elsevier. (**d**) Schematic diagram of the fabrication process for GGFB and GGFF via chemical vapor deposition. (**e**) Scanning electron microscopy image of GGFF with a fiber diameter of approximately 7 μm. Scale bar is 1 mm. (**f**) Simplified schematic diagram of the heat transfer process in the graphene multilayer structure GGFF system. In the figure, 1 and 2 represent the enhanced radiative processes resulting from the increase in graphene layers, 3 and 4 denote the heating process of the underlying glass fiber through thermal radiation and thermal conduction by graphene, and 5 signifies the process wherein the heated glass fiber, together with graphene, acts as an infrared emitter to radiate energy into the surrounding environment. (**d**–**f**) reproduced with permission from [105]. Copyright (2022) American Chemical Society. (**g**) Fabrication process and structural characteristics of the meta-fabric for self-sustained radiative heating. (**h**) Typical field emission scanning electron microscopy images of PMMA fibers with different WF contents. (**i**) Temperature differences in skin simulators covered by different fabric samples at the same location. CB is carbon black, EFM is electrospun fiber membrane, AFM is aerogel fiber membrane, TU is thermal underwear, H-TU is heated thermal underwear, TR is thermal resistance, MIR is mid-infrared. (**g**–**i**) reproduced with permission from [106]. distributed under the terms of the Creative Commons Attribution License (CC BY 4.0).

In contrast to the radiative cooling effect, increasing the mid-infrared reflectivity of fibers can enable radiative heating functionality, thereby reducing heat loss. Radiative heating offers benefits such as high thermal efficiency, no need for a medium, and good penetration capability. Conventional organic materials and textiles naturally exhibit high emissivity, so low-emissivity materials are needed to improve the surface radiative properties of fibers. Li et al. [104] employed thermoplastic polyurethane (TPU), comprising soft and hard segments, as a spinning matrix to fabricate TPU fibers with a uniform porous architecture through controlled phase separation diffusion. Subsequently, they constructed Ti_3_C_2_T_x_ (MXene)-modified fibers (MPHPUF) via layer-by-layer assembly of MXene on the surface of HPUF (refer to Figure 8a,b). These MPHPUF fibers exhibit high mid-infrared reflectivity and solar absorption properties, with an average reflectivity of 79% within the 7–14 μm wavelength range, substantially surpassing conventional cotton and polyester textiles. At a skin temperature of 35 °C, the MPHPUF textiles attain 39.4 °C, and their elevated mid-infrared reflectivity results in a 4.4 °C increase in simulated skin temperature, demonstrating exceptional passive radiative heating capabilities (see Figure 8c). Another methodology involves surface modification of standard fibers with metallic nanomaterials to enhance reflectivity. Yuan et al. [105] developed a flexible dual-emissive infrared electrothermal fiber, designated as graphene-glass fiber (GGF), through chemical vapor deposition (CVD) (refer to Figure 8d). GGF is produced by growing graphene layers on glass fibers using CVD. The radiative characteristics of the graphene layer, along with the high infrared emissivity of glass fibers and the alignment of emission and absorption bands, enable both components to function as infrared emitters, thereby augmenting the fiber’s infrared radiation. The gray-body radiation properties of GGF allow modulation of its emission spectrum through temperature control to suit various absorption conditions. A large-area fabric composed of GGF (GGFF) was fabricated using GGF as the substrate (see Figure 8e), exhibiting wavelength-independent high infrared emissivity (92%) and thermal radiation efficiency (79.4%). A simplified schematic illustrating the infrared heating mechanism of GGFF is presented in Figure 8f. GGFF demonstrates superior radiative energy efficiency compared to conventional alloy heating wires, achieving a 33.3% energy saving. GGF holds significant potential to advance energy-efficient thermal management. Tian et al. [106] developed an aerogel-structured micro/nano-fiber meta-fabric capable of self-heating via radiation (refer to Figure 8g). Utilizing a dual-aerogel strategy, they embedded carbon black nanoparticles (CBNPs) into PMMA fiber pores through humidity-induced electrospinning (see Figure 8h), thereby directly forming a three-dimensional interconnected network meta-fabric. The CBNPs are uniformly distributed within the fibers, absorbing near-infrared radiation from both the human body and the environment. The interconnected nanopores enhance radiation scattering and absorption, working synergistically with the Anderson localization effect of CBNPs to improve heat retention. This composite fabric exhibits a low thermal conductivity of 15.8 W·m^−1^·K^−1^ and can sustain a temperature increase of 8.8 °C (see Figure 8i).

**Figure 9:**
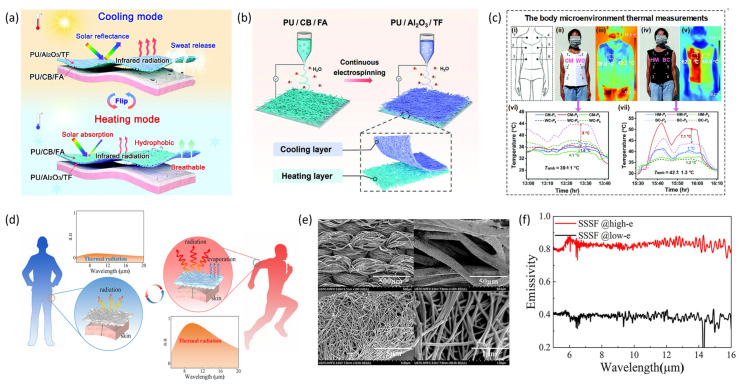
Environmentally Adaptive Radiative Thermal Regulation Functional Fibers. (**a**) Design principle and working mechanism of the dual-mode LNT. (**b**) Schematic diagram of the continuous electrospinning process for fabricating LNT. (**c**) Outdoor thermal measurements, practical wearing tests, and potential applications of the dual-mode LNT, demonstrating the microclimate between the human body and clothing (Shanghai, China, 18 September 2023). (**i**) Illustrates six upper-body measurement sites (P1–P6) designated as temperature monitoring points, (**ii**) demonstrates data acquisition using iButton temperature sensors affixed to the skin surface, (**iii**) presents infrared imaging results showing the LNT outer surface temperature being 2.5 °C lower than white cotton fabric after 40 min of sunlight exposure while worn by a volunteer, (**iv**) depicts the protocol of vest replacement for radiative heating performance evaluation, (**v**) reveals infrared thermography indicating significantly higher temperature on the LNT heating side (62.7 °C) compared to black cotton fabric (49.9 °C), yielding a ≈12.8 °C differential, (**vi**) documents maximum regional temperature differences during cooling tests: ≈8.0 °C at chest (P2), ≈4.1 °C between P6 and P3, and 1.6 °C between P4 and P1, (**vii**) demonstrates that during heating tests under afternoon low-intensity sunlight, the LNT-covered chest region (P2) maintained the highest recorded temperature of 53.1 °C, exhibiting ≈7.1 °C superiority over black cotton fabric, with P1/P4 and P3/P6 differentials measuring 1.0 °C and 1.2 °C respectively. (**a**–**c**) reproduced with permission from [107]. Copyright (2024) Wiley. (**d**) Schematic illustration of the thermal management of SSSF during human body rest and physical activity states. (**e**) Scanning electron microscopy image of the SSSF. (**f**) Spectral emissivity of the SSSF under different operating modes. (**d**,**e**) reproduced with permission from [108]. distributed under the terms of the Creative Commons Attribution License (CC BY 4.0).

Although considerable advancements have been achieved in the investigation of radiative cooling and radiative heating fibers, materials possessing a singular spectral characteristic are incapable of fulfilling thermal requirements under both cold and hot environmental conditions. Consequently, there is an emerging necessity for radiative functional fibers that can facilitate bidirectional thermal regulation. Recently, Cheng et al. [107] created a breathable, biomimetic leather-like nanotextile (LNT) featuring asymmetric wrinkled photonic microstructures and Janus wettability via a single-step electrospinning process (Figure 9b) to enhance personal thermal regulation. The LNT was fabricated by self-adhering a hydrophilic cooling layer to a fiber network, which was welded to a hydrophobic photothermal layer, forming a dual-layered wrinkled structure with exceptional optical properties, wettability gradient, and distinctive texture. This configuration enables solar reflection to reduce heat absorption during cooling mode and solar absorption to increase heating mode, as illustrated in Figure 9a. The LNT achieves effective cooling (22.0 °C) and heating (22.1 °C) under sunlight, expanding the thermal management range by 28.3 °C relative to conventional textiles (Figure 9c). Li et al. [108] introduced a spectrally adaptive smart fabric (SSSF) (Figure 9d) by coating highly breathable polyurethane fabric with a silver nanowire network (Figure 9e). The fabric’s emissivity passively adapts based on its wet or dry state, dynamically regulating radiative heat dissipation. Under dry conditions, the SSSF maintains a low-emissivity state (*ρ* = 0.39) to minimize heat loss, whereas in wet conditions, it shifts to a high-emissivity state (*ρ* = 0.83), thereby enhancing radiative cooling (Figure 9f). Testing indicated that in the low-emissivity mode, the SSSF reduces heat loss by 19.5% compared to conventional fabrics. Conversely, in the high-emissivity mode, the cooling capacity for simulated skin increases by 67.6%. The SSSF effectively modulates human heat dissipation under various conditions, providing superior adaptive thermal management. Hsu et al. integrated a dual-mode infrared emitter within infrared-transparent NanoPE, featuring a high-reflectivity copper layer and a high-emissivity carbon layer. With the low-emissivity layer (0.303) oriented outward, the fabric’s temperature elevates to 40.3 °C, facilitating radiative heating. Conversely, when the high-emissivity layer (0.894) faces outward, the temperature decreases to 33.8 °C, promoting radiative cooling.

As a passive thermal management technology, thermal radiation shows significant potential in personal and building thermal regulation, wearable electronics, and energy harvesting and conversion. By artificially adjusting the surface emissivity, absorptivity, and reflectivity of materials, three types of thermal regulation—cooling, insulation, and heating—can be achieved. Among these, radiative cooling is the main focus in radiative thermal management research. Polyolefin materials, especially polyethylene (PE), exhibit high transparency to radiation within the mid-infrared wavelength range. Fibers based on PE demonstrate excellent radiative cooling performance. Additionally, cooling can be achieved by increasing reflectivity through the incorporation of high-reflectivity nanoparticles or the development of microporous structures, while improving surface emissivity is commonly done through the design of photonic structures. Radiative heating, on the other hand, is achieved by raising surface infrared reflectivity. Metal materials, modified fibers with metallic nanomaterials, MXene, and graphene are among the key materials in passive radiative heating research. Despite notable progress, current radiative thermal regulation materials still face significant limitations. First, single-function radiative thermal regulation products cannot meet the diverse thermal management needs across different scenarios. Second, environmental factors often impact the practical deployment of radiative materials, causing actual thermal regulation efficiency to fall short of expectations. Future development should focus on creating adaptable materials capable of meeting various thermal regulation needs, dynamic radiative management systems that can switch modes in response to ambient temperature changes, and multifunctional composite smart fibers.

## 3. Thermal Energy Storage Functional Fibers

Phase change materials (PCMs) with high latent heat can absorb or release thermal energy during phase transitions, thereby reducing significant fluctuations in ambient temperature [109], making them a promising class of passive thermal management materials. Based on material composition, PCMs can be categorized into organic, inorganic, and eutectic mixtures [110]. Among these, organic materials have gained more research attention due to their wide phase transition temperature range, high energy storage density, and the lack of supercooling and phase separation phenomena compared to inorganic materials [111]. PCMs are commonly used in temperature regulation applications such as electronic chip cooling [112] and thermal protection in extreme environments [113].

Thermal energy storage fibers refer to a novel functional fiber system that incorporates sensible heat, latent heat, or thermochemical energy storage units at the fiber scale, enabling reversible and controllable thermal energy absorption and release while maintaining characteristics such as flexibility, weavability, and high specific surface area. These fibers can stabilize the internal temperature environment of a system within a certain temperature range and are thus termed intelligent temperature-regulating fibers. Compared to traditional thermal storage materials, the fiber-based design significantly enhances specific surface area and heat transfer rates, facilitating integration into various forms such as textiles, felts, and composite reinforcements, which can be seamlessly incorporated into clothing, insulation felts, filtration media, or composite structures. Consequently, such materials exhibit outstanding engineering value in industrial applications, including high-temperature pipeline and equipment insulation, waste heat recovery felts, and thermal management-enhanced composites, thereby improving process energy efficiency and mitigating temperature fluctuations. In the field of personal thermal management, thermal storage fibers can maintain the thermal stability of the human microenvironment under extreme conditions, such as in spacesuits, firefighting gear, and mountaineering apparel [114,115]. In the electronics sector, they serve as thermal protection materials for batteries and integrated circuits [112]. Additionally, thermal storage fibers demonstrate considerable potential in emerging flexible wearable devices and sensors [116]. With the increasing emphasis on carbon neutrality and the efficient utilization of industrial waste heat, thermal energy storage fiber materials are expected to become a critical node in multi-scale thermal management, bridging the gap between “material-structure-system” and providing practical solutions for industrial energy conservation, renewable energy utilization, and comfort-oriented environmental control. Due to the unique structure and application scenarios of thermal storage fibers, the selected phase change materials (PCMs) generally exhibit high chemical stability, excellent thermal conductivity, strong processability, and high energy storage density. Commonly used PCMs in thermal storage fibers include inorganic materials such as hydrated salts, molten salts, and metals; organic materials such as acid esters, paraffin wax, and polyols [117,118]; and composite phase change materials. The composite design strategies for thermal storage fiber materials can be categorized as follows: hollow fiber filling, phase change material coating, sheath-core composite spinning, and phase change material capsule blending.

**Figure 10:**
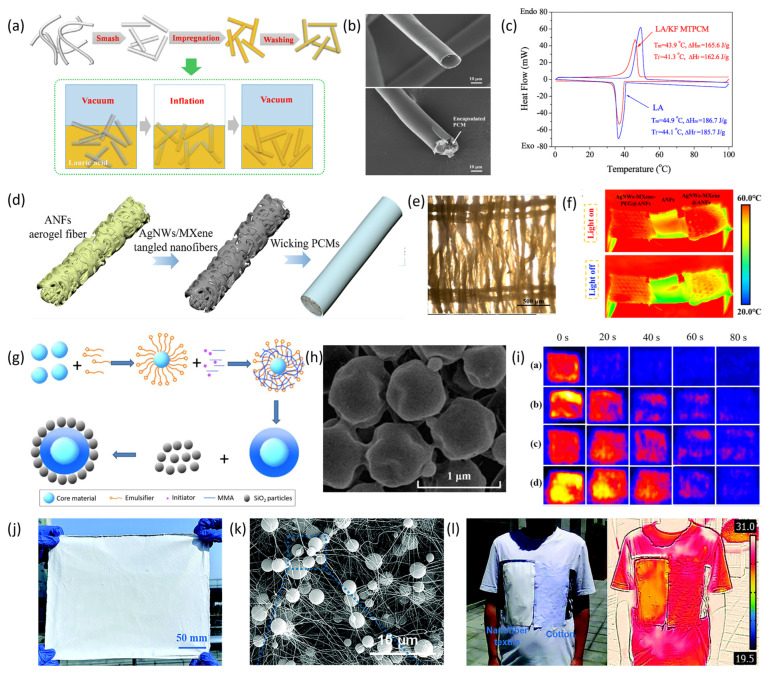
Phase-change thermal storage fiber fabricated by hollow filling and microcapsule composite spinning. (**a**) Schematic diagram of the preparation of MTPCM by vacuum impregnation. (**b**) FE-SEM images of KF and LA/KF MTPCM. (**c**) Differential scanning calorimetry (DSC) curves of LA and LA/KF microcapsule phase-change materials. (**a**–**c**) reproduced with permission from [120]. Copyright (2019) Elsevier. (**d**) Schematic diagram of conductive aerogel fiber with a three-dimensional hierarchical porous structure for constructing intelligent multi-responsive fibers and self-powered textiles. (**e**) Optical photograph of ANFs-based fabric woven from aerogel fibers. (**f**) Infrared images of ANFs, AgNWs-MXene@ANFs, and AgNWs-MXene-PEG@ANFs fabrics under a 60 °C ambient background temperature after removal of light stimulation (10 s). (**d**–**f**) reproduced with permission from [121]. Copyright (2022) Elsevier. (**g**) Schematic diagram of the preparation of PCM microcapsules using PMMA and SiO_2_ particles as wall materials. (**h**) SEM image of MCs1 microcapsules. (**i**) Infrared thermal images of denim fabrics treated with different microcapsules after 80 s of tungsten lamp irradiation. (a) Original fabrics, (b) fabric coated with PA, (c) fabrics coated with MCs1, (d) fabrics coated with MCs2. (**g**–**i**) reproduced with permission from [126]. Copyright (2020) Elsevier. (**j**) Hierarchical nanofiber fabric embedded with a large number of phase-change microcapsules (PCMC) prepared by electrospinning. (**k**) SEM images of both surfaces of the nanofiber fabric. (**l**) Photographs and infrared thermal images of the nanofiber fabric and cotton fabric worn on the human chest upon reaching thermal equilibrium. (**j**–**l**) reproduced with permission from [128]. Copyright (2024) Wiley.

The hollow fiber filling method involves encapsulating phase change materials (PCMs) within the hollow cavities of fibers. Typically, hollow fiber blocks or membranes are first prepared, followed by impregnating them with liquid PCMs through physical adsorption to create fibers with thermal energy storage capabilities. Vigo and Frost et al. [119] were among the first to propose using hollow fibers to make phase change fibers. They encapsulated hydrated inorganic salts—a type of PCM containing crystalline water—into hollow fibers, developing fibers with heat storage functions. However, after several cycles of heating and cooling, the inorganic salts tended to leach out from the fibers, causing a decline in thermal storage performance. Song et al. [120] developed microtube-encapsulated phase change materials (MTPCMs) by embedding lauric acid (LA) into hollow natural kapok fiber microtubes via a straightforward vacuum impregnation process (see Figure 10a,b). These MTPCMs demonstrated a high thermal energy storage capacity, achieving 87.5% of the capacity of pure LA. They also exhibited exceptional cycling stability, maintaining their thermal storage performance without significant degradation after 2000 continuous charge–discharge cycles. Meanwhile, Zuo et al. [121] developed an intelligent aramid aerogel fiber with dual functions for energy storage and conversion. They infused polyethylene glycol (PEG) into wet-spun porous aramid aerogel fibers and coated them with transparent fluorosilicone (FSi) resin (see Figure 10d,e). The hierarchical porous structure within the aerogel fibers generated strong capillary forces and confinement effects for PEG, whose broad phase transition temperature range and high latent heat rendered the fibers effective for phase change thermal energy storage (see Figure 10f).

Phase change material coating is a technique that gives traditional fibers the ability to store thermal energy by applying phase change materials to their surfaces. Vigo et al. [122] combined polyethylene glycol, crosslinking agents, and catalysts to create a uniform aqueous solution. Fabrics like cotton, polyester-cotton blends, and wool were soaked in this solution, then pressed, dried, and washed, creating composite fibers that showed significant heat absorption and release within the temperature range of 0 °C to 50 °C. However, fibers with thermal storage created through direct coating methods tend to have poor wash resistance and limited durability of the phase change materials. Additionally, since the phase change materials are unencapsulated, they are highly susceptible to leakage.

The microencapsulated composite spinning method involves encapsulating phase change materials (PCMs) within a specific temperature range using physical or chemical techniques to create stable micro- or nano-scale capsule particles. These PCM-containing capsules are then incorporated into fibers through blending and spinning processes, forming composite fibers with thermal energy storage capabilities. Common methods for preparing such composite fibers include wet spinning [123], electrospinning [124], and melt spinning [125]. The encapsulation effectively prevents PCM leakage, while the composite spinning technique ensures even dispersion of PCM capsules within the fiber, leading to consistent and reliable thermal storage performance. Zhang et al. [126] proposed a method for creating phase-change thermal storage textiles by directly applying PCM capsules onto denim fabric. They employed a two-step process to produce PMMA/SiO_2_ PCMMCs (see Figure 10g), wherein the phase-change material was a blend of paraffin and butyl stearate, with PMMA functioning as the capsule coating (see Figure 10h). As depicted in Figure 10i, the denim fabric coated with PCM capsules demonstrated better temperature regulation compared to uncoated fabric. Li et al. [127] prepared phase-change microcapsules using RT27 paraffin (PW) as the core material and SiO_2_ derived from tetraethyl orthosilicate as the shell via in situ polymerization. These microcapsules were uniformly dispersed into polyvinyl alcohol (PVA) to form a spinning dope, which was subsequently processed into PW/PVA phase-change fibers through wet spinning. The microcapsules had an average size of 1.39 μm and were homogeneously distributed within the fibers. The phase-change energy storage fibers achieved a PW encapsulation efficiency of 94.72%, indicating high encapsulation quality, with a phase-change enthalpy of 45.39 kJ/kg. Gu et al. [128] developed a hierarchically structured nanofiber textile embedded with a high content of phase-change microcapsules (PCMCs) via electrospinning (see Figure 10j). The textile comprised a PVDF-HFP fiber layer and a PVB/PCMC60 fiber layer doped with 60 wt% PCMCs, as illustrated in Figure 10k. The PVB/PCMC-60 layer functioned as the thermal energy storage and temperature regulation component. The substantial loading of PCMCs endowed the nanofiber textile with a significant heat storage capacity of 92.6 J/g. Compared to PVDF-HFP/PVB and cotton, this nanofiber textile exhibited a temperature decrease of 10.1 °C (see Figure 10l), demonstrating that the incorporation of the PCMC layer markedly enhanced its cooling performance.

**Figure 11:**
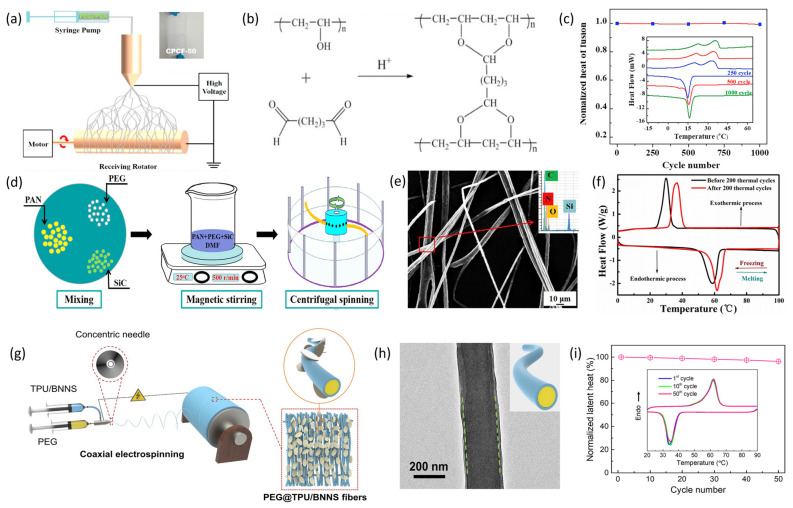
Direct encapsulation of phase change materials for constructing functional heat storage fibers. (**a**) Schematic diagram of the preparation of novel PEG/PVA composite phase change fibers via electrospinning. (**b**) Schematic diagram of the chemical cross-linking mechanism of PEG/PVA composite phase change fibers. (**c**) Normalized latent heat of melting for CPCF-50 during the first heating cycle (inset shows the corresponding DSC cycle curve). (**a**–**c**) reproduced with permission from [129]. Copyright (2021) Elsevier. (**d**) Schematic diagram of the preparation of PAN/PEG/SiC phase change materials via centrifugal spinning. (**e**) SEM image of physically cross-linked PAN/PEG/silicon carbide (SiC) phase change material fibers, with the corresponding energy-dispersive X-ray spectroscopy (EDS) spectrum. (**f**) Differential scanning calorimetry curves of PAN/PEG/SiC phase change fibers with 4.0 wt% SiC before and after 200 thermal cycles. (**d**–**f**) reproduced with permission from [131]. Copyright (2020) Elsevier. (**g**) Schematic diagram of the preparation of PEG@TPU/BNNS core–shell structured nanocomposite fibers via electrospinning. (**h**) TEM image of PEG@TPU fibers, with the inset in the upper right corner showing a schematic diagram of the corresponding core-sheath fiber structure.The green dashed line delineates the boundary between the core and shell layers. (**i**) Normalized latent heat of melting for PEG@TPU/BNNS-es with approximately 32 wt% BNNSs, with the inset showing the DSC curves for the 1st, 10th, and 50th thermal cycles of the material. (**g**–**i**) reproduced with permission from [132]. distributed under the terms of the Creative Commons Attribution License (CC BY 4.0).

Besides the microencapsulated PCM composite spinning method, another approach involves directly encapsulating PCM within the fiber structure. This method incorporates phase change materials, which are often difficult to spin, into the spinning matrix through physical dissolution or chemical cross-linking grafting, followed by direct spinning. Alternatively, fibers can be constructed with a “core–shell” structure to encapsulate PCM and create phase change composite fibers. These fibers generally show better leakage prevention, and textiles made from them offer improved flexibility and breathability. Zhu et al. [129] introduced a novel PEG/PVA composite phase change fiber. By applying a simple surface cross-linking technique, PEG was cross-linked onto the molecular chains of the PVA fiber matrix, and its aqueous solution was subsequently electrospun to produce a new PEG/PVA phase change composite nanofiber (see Figure 11a). As illustrated in Figure 11b, due to the chemical cross-linking between PEG and PVA, no PEG leakage occurred during long-term use of the phase change composite nanofibers. The CPCF-50 composite fiber displayed a regular morphological structure and had a latent heat of 72.3 J/g (see Figure 11c). Bao et al. [130] employed a wet-spinning method to fabricate a diameter-controllable smart hydrophobic Kevlar aerogel fiber (PW@HKAF) for encapsulating paraffin wax (PW). Initially, hydrophobic Kevlar aerogel fibers were produced via wet-spinning, followed by immersion in molten paraffin wax to adsorb the phase change material, resulting in the formation of PW@HKAF. Due to the porous structure of the aerogel fibers, characterized by high porosity and a large specific surface area, the strong capillary forces within the pores effectively confine the paraffin wax inside the fiber pores, thereby preventing leakage. Subsequently, the phase change fibers were woven into flexible textiles, demonstrating a high latent heat of approximately 135.1–172 J/g. In addition to electrospinning, centrifugal spinning serves as an alternative method for fabricating encapsulated phase change bi-component fibers. Compared to electrospinning, centrifugal spinning offers advantages such as higher production efficiency, safer operation, and lower requirements for the spinning solution. Chen et al. [131] utilized a straightforward centrifugal spinning approach to prepare polyethylene glycol (PEG)-based phase change material fibers (PAN/PEG/SiC PCM fibers), employing polyacrylonitrile (PAN) as the supporting material and N, N-dimethylformamide as the solvent for polymer dissolution (see Figure 11d). These fibers physically combine the various materials without chemical reactions, as illustrated in Figure 11e. The PAN support effectively restricts leakage of the PEG phase change material. Experimental results indicated that after 200 heating-cooling cycles, the fibers maintained a stable shape without leakage. The PCM fibers melted at 51.31 °C with a latent heat of 69.91 J/g and froze at 33.71 °C with a latent heat of 66.68 J/g (see Figure 11f). The PAN/PEG/SiC PCM fibers demonstrated favorable thermophysical properties, including reliability and stability, indicating their potential for long-term applications. Besides constructing physicochemical cross-linked structures within the fibers to prevent PCM leakage, another strategy involves employing coaxial electrospinning to produce multicomponent fibers with a “core–shell” structure, where the PCM is encapsulated within the hollow core of the fiber to prevent leakage. Lin et al. [132] fabricated oriented phase change nanofibers (PCN) with a core-sheath structure via coaxial electrospinning, as illustrated in Figure 11g. The sheath layer of the composite fiber comprised thermoplastic polyurethane (TPU) and boron nitride nanosheets (BNNS), whereas the core layer consisted of polyethylene glycol (PEG) phase change material, as depicted in Figure 11h. The film produced by hot-pressing the PCN fibers demonstrated a high fracture strain of 45% and a relatively elevated phase change enthalpy of 101 J/g. Experimental results indicated that, after 50 melting-solidification cycles, the differential scanning calorimetry (DSC) curve of the PCN fibers nearly overlapped with the initial curve (see Figure 11i), indicating high stability and no leakage. These fibers exhibit broad potential applications in thermal management for 5G base stations. Kou et al. [133] employed coaxial electrospinning to develop composite temperature-regulating fibers with a core–shell architecture. The composite fiber utilized polyacrylonitrile (PAN) as the shell and n-octadecane (PW) phase change material as the core. The composite fiber membrane exhibited a high melting enthalpy of 171.6 J/g, with thermal energy storage and release temperatures concentrated within the range of human comfort, rendering it suitable for applications in smart textiles and personal thermal regulation. Zhu et al. [134] reported a fiber membrane intended for personal thermal management employing radiative cooling-phase change (RC-PC). The fibers, fabricated by coaxial electrospinning, possessed a core–shell configuration. The shell comprised poly(3-hydroxybutyrate-co-3-hydroxyvalerate) (PHBV)/TEOS prepolymer, while the core contained n-octadecane as the phase change material. The PHBV/TEOS shell served as the carrier for radiative cooling functionality, and n-octadecane facilitated thermal energy storage through phase change. The bi-component composite fibers demonstrated a phase change enthalpy of 88.3 J/g. Following repeated washing and 50 thermal charge–discharge cycles, no significant reduction in phase change enthalpy was observed, indicating that the core–shell structure and internal pore architecture effectively prevented leakage of the phase change material. In summary, from the perspective of encapsulation methods, nano-confinement exhibits diverse encapsulation forms and comparatively effective encapsulation outcomes relative to other techniques. Regarding fabrication methods, composite spinning provides superior fiber controllability, while blend spinning offers higher flexibility in preparation compared to alternative approaches. The integrated application of various encapsulation strategies and fiber fabrication techniques facilitates the production of target fibers with exemplary performance.

Currently, considerable advancements have been achieved in the design and manufacturing processes of thermal energy storage fibers; however, numerous challenges persist in their practical implementation. Firstly, while strategies such as microencapsulation technology and composite spinning with phase change materials can significantly mitigate leakage, the complete prevention of liquid phase change material leakage remains a formidable challenge. Consequently, some researchers have proposed the utilization of solid–solid phase change materials as an alternative to solid–liquid phase change materials, with the aim of fundamentally eliminating leakage risks. Liang et al. [135] reported a supramolecular solid–solid phase change polyethylene glycol (ScPEG) coating based on multiple hydrogen bonding interactions, employed to encapsulate silver nanowire (AgNW)-decorated glass fiber fabric (A-GFF). The ScPEG was synthesized through hydrolysis-condensation of polyethylene glycol with active end groups, preserving the phase change properties of the original PEG while demonstrating excellent shape stability. Polyethylene glycol molecular chains are capable of storing and releasing heat by transitioning between crystalline and amorphous states. Liu et al. [136] developed a flexible solid–solid phase change fiber (PCF)—polyethylene glycol/4,4′-methylenebis(cyclohexyl isocyanate) fiber—via polycondensation and wet-spinning techniques. By combining HMDI with PEGs of various molecular weights through wet-spinning, phase change material fibers (PMFs) exhibiting solid–solid phase transitions were fabricated. The phase change temperature and enthalpy of the PMFs could be modulated by adjusting the molecular weight of PEG. The resulting PMFs demonstrated high mechanical strength and excellent thermal cycling stability, with the phase change enthalpy remaining nearly unchanged after 2000 heating-cooling cycles.

Additionally, since phase change materials with high heat storage capacity and the fiber matrix itself are organic materials with poor thermal conductivity, the overall thermal conductivity of phase change composite nanofibers remains relatively low. Extremely low thermal conductivity can reduce the heat exchange efficiency of the fibers, thus affecting their ability to store and release heat, which limits the application of heat storage fibers [137,138]. A common approach to enhance the overall thermal conductivity of fibers is to add thermally conductive fillers. Frequently used fillers include boron nitride nanosheets, graphene nanosheets, metals, and highly thermally conductive ceramic particles such as silicon carbide and aluminum oxide [139,140]. Lin et al. [132] showed that coating phase change bi-component composite fibers (PEG@TPU), prepared via coaxial electrospinning, with a highly thermally conductive network of hexagonal boron nitride nanosheets (BNNS) through electrostatic spraying, resulted in a PCN film that, after hot pressing, achieved a relatively high in-plane thermal conductivity (28.3 W/m·K) with a low BNNS loading of 32 wt%. Furthermore, introducing BNNS had no significant effect on the latent heat of the bi-component composite fibers. Hao et al. [141] introduced an innovative microfluidic approach for encapsulating phase change materials within flexible fibers. By employing this technique, liquid paraffin RT25 was encapsulated within a polyvinyl butyral (PVB) shell to fabricate phase change fibers with a core–shell architecture. Furthermore, graphene was coated onto the surface of the fibers through pneumatic spraying to produce graphene-coated phase change fibers (GPCFs). The application of microfluidic technology allows for precise regulation of fiber dimensions and composition, while the graphene coating significantly enhances the thermal conductivity and infrared emissivity of the fiber surface. These multicomponent composite fibers demonstrate considerable potential for thermal management in periodic, short-term, high-power electronic devices. Chen et al. [131] prepared phase change material fibers containing nano-silicon carbide (SiC) fillers via centrifugal spinning. The fibers were directly produced from a mixture of polyacrylonitrile (PAN), polyethylene glycol (PEG), and SiC, with physical interactions among the constituents. PAN served as the supporting matrix, PEG functioned as the phase change thermal storage material, and SiC was incorporated as a high-thermal-conductivity filler to improve the thermal conductivity of the multicomponent composite fibers. The inclusion of SiC increased the thermal conductivity by 35%, reaching 0.334 W/m·K relative to the unmodified fibers. Moreover, the addition of SiC contributed to enhanced shape stability of the fibers.

Phase-change composite fibers, also referred to as intelligent temperature-regulating fibers, constitute a category of passive thermal management fibers, Table 3 summarizes the materials and properties of various phase-change composite fibers. By employing strategies such as hollow fiber filling, surface coating, microcapsule composite spinning, surface cross-linking technology, and core–shell encapsulation, phase change materials (PCMs) with thermal energy storage capabilities are integrated into conventional fibers, thus producing composite phase-change thermal storage fibers with adaptable temperature-regulating functions. The thermal storage substances utilized within these fibers encompass hydrated inorganic salts, molten salts, paraffins, and polyols such as polyethylene glycol. Presently, phase-change composite fibers exhibit extensive potential for applications including personal thermal management under extreme conditions, biomedicine, energy materials, and electronic devices. Concurrently, the development and utilization of these fibers encounter several technical challenges. Foremost among these is the leakage of PCMs, which remains a significant impediment to the advancement of phase-change composite fibers. Although techniques such as microcapsule composite spinning, coaxial electrospinning, and porous adsorption effectively confine and encapsulate PCMs, complete prevention of leakage is not achievable. Additionally, the complexity of encapsulation and spinning procedures elevates production difficulty and manufacturing costs. Secondly, the homogeneity of PCM distribution within the fibers cannot be entirely assured. Moreover, the thermal storage performance of phase-change composite fiber products tends to diminish notably after repeated usage cycles. Lastly, the existing methods for preparing composite fibers generally feature low efficiency and high costs, rendering large-scale production and application still beyond immediate reach. To overcome these challenges, future research may prioritize enhancements in nanofiber fabrication techniques and PCM encapsulation technologies, as well as the development of solid–solid phase-change composites to mitigate leakage issues. In order to improve filler uniformity, surface treatment and modification strategies for thermal conductive fillers can be advanced to produce composite phase-change nanofibers with homogeneous structures and excellent thermal conductivity. Likewise, phase-change composite fibers may be integrated with other functional fibers to facilitate the design and application of innovative multifunctional composite fibers.

## 4. Thermal Energy Converting Functional Fibers

The previous discussion focused on the regulation of heat transfer processes within the system, achieving passive thermal management through both heat transport and heat storage approaches. These two methods solely manipulate and utilize thermal energy as a single form of energy. However, nature encompasses various other forms of energy, such as light energy, electrical energy, and mechanical energy, which can be converted into thermal energy through physical or chemical means. The transformation between energy forms not only enables the system to simultaneously harvest and utilize multiple energy forms but also fundamentally expands the spatial dimensions and technical pathways of thermal management.

At the fiber scale, thermal functional fibers can be categorized into several types based on their dominant energy conversion pathways, enabling reversible or unidirectional transformations between thermal energy and electrical energy, light energy, mechanical energy, etc. Firstly, in terms of the mutual conversion between thermal and electrical energy, thermal functional fibers can be classified into two material types based on thermoelectric and Joule heating effects: thermoelectric fibers and Joule heating fibers. The former holds significant application value in personal thermal management, self-powered wearable devices, and low-grade waste heat recovery in industrial and architectural settings. The latter has been widely utilized for localized and zonal temperature regulation in scenarios such as human body, transportation, and buildings. Secondly, photothermal functional fibers, constructed via the photothermal effect, can efficiently convert light energy into thermal energy, demonstrating outstanding application potential in fields such as solar-driven seawater desalination. Thirdly, thermally actuated functional fibers, relying on shape-memory materials or multilayer thermal expansion mismatch structures, can generate controllable mechanical responses upon heating, converting thermal energy into mechanical energy. These fibers provide critical material support for emerging applications such as soft robotics and smart textiles.

### 4.1. Thermoelectric Fibers

Thermoelectric materials possess the capability to convert thermal energy directly into electrical energy and vice versa, based on the thermoelectric effect [143]. Conventionally, flexible thermoelectric devices are predominantly manufactured from bulk materials, thin films, or substrate-based thermoelectric materials [144]. However, these two-dimensional planar flexible structures frequently encounter challenges in effectively utilizing temperature gradients along the thickness direction [145]. Moreover, thin-film configurations are associated with limitations such as poor breathability and restricted two-dimensional bending flexibility, which can diminish comfort when employed in wearable electronic devices [146]. Fibers, as one-dimensional materials characterized by high aspect ratios, offer distinct advantages including three-dimensional flexibility, lightweight properties, and ease of integration within textiles. Consequently, thermoelectric fibers demonstrate significant potential for broad applications in personal thermal management, the thermal regulation of electronic devices, and flexible wearable thermoelectric systems [147,148,149].

The fundamental principle by which thermoelectric fibers achieve thermoelectric energy conversion is the thermoelectric effect (TE), which comprises two basic effects: the Seebeck effect and the Peltier effect [150]. The Seebeck effect, known as the primary thermoelectric effect, refers to the phenomenon where a temperature difference applied across a thermoelectric material causes charge carriers within the material to migrate from the hot end to the cold end, thereby generating an electric potential difference between the two ends. This principle underlies thermoelectric power generation [151]. The Peltier effect, being the inverse of the Seebeck effect, describes how an applied electric potential difference across a thermoelectric material induces a temperature difference between its ends, forming the basis for thermoelectric cooling [152]. The energy conversion efficiency of thermoelectric conductive materials can be quantified by the thermoelectric figure of merit, *ZT* [153], defined as follows:

(6) $$ZT=\frac{s^{2}\sigma T}{\kappa }$$

Here, *S* is the Seebeck coefficient, *σ* is the electrical conductivity, *T* is the thermodynamic temperature, and *K* is the thermal conductivity. According to this formula, increasing the electrical conductivity and Seebeck coefficient while decreasing the thermal conductivity can improve the *ZT* value. However, these three parameters are not completely independent [154]. Therefore, a key challenge in thermoelectric materials is how to decouple the thermoelectric parameters and simultaneously control both thermal and electrical properties to enhance performance [151,152]. Based on the type of thermoelectric materials, thermoelectric fibers are categorized into inorganic thermoelectric fibers (such as Bi_2_Te_3_, PbTe, SnSe, metal oxides, etc.), organic thermoelectric fibers (such as polyaniline (PANI), polyacetylene (PA), polypyrrole (PPy), etc.), and composite thermoelectric fibers.

**Figure 12:**
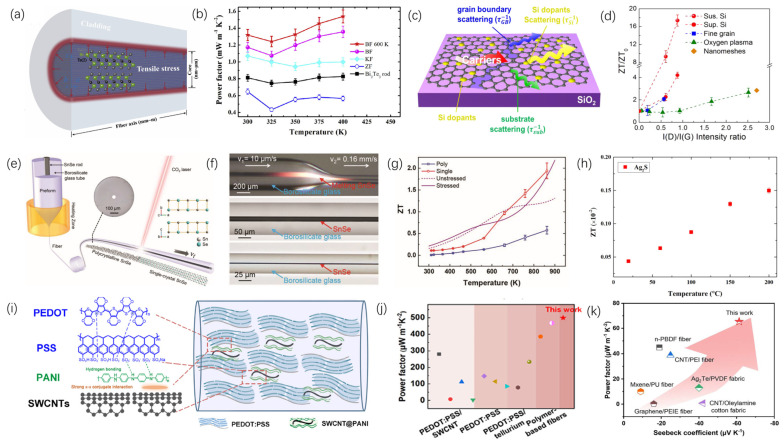
Thermoelectric functional fibers. (**a**) Schematic HR-TEM structure of the BF core. (**b**) Power factor of Bi_2_Te_3_ rods and the fiber core in the temperature range of 300–400 K. (**a**,**b**) reproduced with permission from [157]. Copyright (2020) Elsevier. (**c**) Schematic illustration of energy carrier transport scattering factors in supported silicon-based graphene. (**d**) Normalized thermoelectric conversion efficiency corresponding to different types of defects in monolayer chemical vapor deposition graphene. (**c**,**d**) reproduced with permission from [161]. Copyright (2021) Elsevier. (**e**) Schematic of the thermal drawing and subsequent laser recrystallization process for SnSe fibers. (**f**) Diameter-tunable single-crystal SnSe core fiber fabricated based on the CO_2_ laser tapering process. (**g**) Thermoelectric performance of SnSe fibers. (**e**–**g**) reproduced with permission from [158]. Copyright (2020) Wiley. (**h**) *ZT* value of Ag_2_S fibers. reproduced with permission from [158]. Copyright (2022) AIP Publishing. (**i**) Schematic of the interaction mechanism in PEDOT: PSS/SWCNT@PANI composite fibers. (**j**) Comparison of the power factor of PEDOT:PSS/SWCNT@PANI composite fibers with literature-reported performance of PEDOT:PSS-based composite fibers. (**i**,**j**) reproduced with permission from [166]. Copyright (2025) Wiley. (**k**) Comparison of the power factor of PC-Ag_2_Te-87.5 with other reported PEDOT:PSS-based multicomponent composite fibers and textiles. reproduced with permission from [168]. Copyright (2025) American Chemical Society.

At room temperature, the majority of inorganic thermoelectric materials demonstrate superior thermoelectric properties relative to other materials [155]. Nonetheless, the intrinsic limitations of inorganic substances, such as elevated cost and limited flexibility, restrict their application in the development and design of fiber-based flexible wearable devices. However, innovative fiber fabrication techniques, including thermal drawing and coating, have introduced new opportunities for inorganic thermoelectric fibers. Common inorganic thermoelectric materials encompass Bi_2_Te_3_, SnSe, AgSe, and PbTe, among others. Tellurium-based thermoelectric systems are among the most commercially established [156]. Specifically, Bi_2_Te_3_ exhibits a high Seebeck coefficient, low thermal conductivity, and high electrical conductivity at room temperature, rendering it a highly promising inorganic thermoelectric material. The layered crystal structure of Bi_2_Te_3_ enhances charge transport and facilitates phonon interfacial scattering, thereby augmenting thermoelectric performance. Layered architectures can also be realized in thermoelectric fibers produced via thermal drawing. Sun et al. [157] examined thermally drawn Bi_2_Te_3_ fibers with three distinct internal tensile stresses, employing cladding materials with varying coefficients of thermal expansion (see Figure 12a). Their findings indicate that the internal stress within the fibers optimized the band structure and improved thermoelectric properties. The Bi_2_Te_3_ fibers ultimately achieved a power factor of 1320 μW·m^−1^·K^−2^ and a *ZT* value of 0.76 (illustrated in Figure 12b). SnSe constitutes another inorganic thermoelectric material featuring a layered crystal structure, distinguished by an exceptionally low lattice thermal conductivity and a high *ZT* value. Zhang et al. [158] synthesized thermally drawn single-crystal SnSe fibers through a CO_2_ laser melting recrystallization process, as depicted in Figure 12e,f. The ultralong SnSe fibers demonstrated exceptional thermoelectric performance, attaining a *ZT* value of 2 at 862 K (Figure 12g). Furthermore, it has been documented that sodium doping into SnSe can produce high-performance p-type thermoelectric materials. Moreover, it has been reported that sodium doping in SnSe can produce high-performance p-type thermoelectric materials. Their research revealed that sodium doping activates multiple valence bands, thereby enhancing thermoelectric performance and achieving an average *ZT* of 1.34 within the temperature range of 300–773 K. Additionally, ductile inorganic materials such as Ag_2_S offer promising prospects for flexible wearable devices, combining excellent thermoelectric properties with notable ductility. Lu et al. [159] successfully fabricated Ag_2_S fibers with a *ZT* value of 0.44 × 10^−3^ for the first time utilizing a thermal drawing technique (Figure 12h). The resultant fibers demonstrated favorable plastic deformation at ambient temperature. Carbon-based materials have garnered significant attention owing to their superior mechanical properties, high electrical conductivity, and exceptional flexibility, positioning them as a promising category of thermoelectric materials. Predominantly, these materials include carbon nanotubes (CNTs) and graphene. As thermoelectric materials, their performance is often constrained by their inherently high thermal conductivity. The electrical properties of CNTs vary markedly depending on the folding angle of graphene nanosheets. Chemical doping is routinely employed to modulate the carrier concentration within CNTs, thereby adjusting the thermoelectric parameters of the material. Lee et al. [160] synthesized CNT fibers via a wet-spinning technique. The pristine CNTs were treated with hydrochloric acid and chlorosulfonic acid to eliminate residual metal catalysts and carbonaceous impurities, and the crystalline sidewalls were protonated to facilitate dispersion without defect formation. Subsequently, thermal annealing was conducted to remove residual acid molecules, leading to a substantial enhancement of the power factor to 4322 μW/m·K^2^. Graphene, being a two-dimensional material, can form quasi-one-dimensional structures such as graphene nanoribbons due to their extremely narrow width. Graphene exhibits both high thermal and electrical conductivities. Improving the thermoelectric performance of graphene materials primarily involves maintaining electrical conductivity while reducing thermal conductivity [147]. Doping graphene with other atoms introduces defects that serve as phonon scattering centers, effectively diminishing the material’s thermal conductivity. Importantly, the doped atoms do not compromise the crystal structure of graphene, leaving its electrical conductivity unaffected by these defects [161]. lee et al. [162] synthesized silicon-doped graphene utilizing low-pressure chemical vapor deposition (see Figure 12c), which exhibited superior thermoelectric performance. They selected methoxytrimethylsilane (MTMS) and hexane as liquid precursors and regulated the doping concentration by modulating the precursor pressure. When the silicon doping level reached 2.59%, the resultant thermoelectric performance was seventeen times greater than that of the suspended material (see Figure 12d). Organic thermoelectric materials are distinguished by their low thermal conductivity, remarkable flexibility, lightweight nature, and ease of processing [154]. The primary organic thermoelectric materials consist of conductive polymers such as polyaniline (PANI), polypyrrole (PPy), poly(3,4-ethylenedioxythiophene) (PEDOT), and polythiophenes (PTs). Due to the straightforward manufacturing of fibers using organic materials, these hold considerable promise in the development of wearable thermoelectric devices. However, the electrical conductivity of organic thermoelectric materials remains comparatively low relative to inorganic materials, thereby constraining the enhancement of their thermoelectric efficiency. Among organic thermoelectric materials, PEDOT: PSS has attracted extensive attention owing to its good water solubility, flexibility, facile doping, and chemical stability. Currently, two principal methods exist to augment the electrical conductivity of PEDOT:PSS: the first involves secondary doping via post-treatment with acids, bases, or organic solvents [163], with ethylene glycol (EG), methanol, and ionic liquids being common secondary dopants; the second entails synthesizing organic composite materials by integrating PEDOT:PSS with carbon nanotubes or inorganic substances [164]. Jalili et al. [165] fabricated continuous PEDOT:PSS fibers employing a wet-spinning technique. They demonstrated that pretreating the spinning formulation with polyethylene glycol could optimize the electrical conductivity of the fibers to 264 S cm^−1^. The increased organization of PEDOT:PSS during the spinning process resulted in enhanced redox properties of the fibers. Wen et al. [166] developed an innovative thermoelectric composite fiber featuring a dual-interface structure composed of poly(3,4-ethylenedioxythiophene):poly(styrenesulfonate)/single-walled carbon nanotube @ polyaniline (PEDOT:PSS/SWCNT@PANI). During the synthesis of the composite fiber via wet spinning, a PANI layer was introduced between the SWCNT and PEDOT:PSS, facilitating the formation of multiple dual interfaces within the fiber and resulting in a highly aligned structure, as illustrated in Figure 12i. Notably, the thermoelectric performance was significantly improved, achieving a Seebeck coefficient of 43.5 ± 0.7 μV K^−1^, an electrical conductivity of 2472 ± 23.3 S cm^−1^, and a power factor of 467.8 ± 10.52 μW·m^−1^·K^−2^ (see Figure 12j).

Composite thermoelectric fibers incorporate organic conductive polymer materials with other highly conductive substances, such as carbon nanotubes, inorganic materials, and metals. The resulting composite thermoelectric materials synergize the low thermal conductivity characteristic of polymers with the high electrical conductivity of other constituents, thereby enhancing the overall thermoelectric performance. Additionally, the conductive polymer, serving as the fiber matrix, imparts the composite with excellent flexibility and ease of fiber formation. Predominant methods for preparing fiber-based composite materials include gelation, wet spinning, and solution coating [149,167]. These composite thermoelectric fibers can be classified into two main types: those utilizing carbon-based materials as fillers, and polymer-based composite fibers employing inorganic nanoparticles or nanowires as fillers. Li et al. [168] reported the development of a high-performance n-type organic-inorganic composite thermoelectric fiber (PEDOT:PSS/PC-Ag_2_TeNWs). This fiber was fabricated via wet spinning, featuring inorganic Ag_2_Te nanowires (PC-Ag_2_TeNWs) coated with poly(3,4-ethylenedioxythiophene):poly(styrenesulfonate) (PEDOT:PSS). The organic coating enhances the dispersion of inorganic Ag_2_Te nanowires within the fiber, allowing the content of PC-Ag_2_TeNWs to reach as high as 87.5 wt% (see Figure 12k). The thermoelectric properties of this composite fiber markedly surpass those of previous n-type thermoelectric fibers, demonstrating a Seebeck coefficient of −61.3 μV/K, a power factor of 65.32 μW/m·K^2^, and a figure of merit (*ZT*) of 0.02.

**Figure 13:**
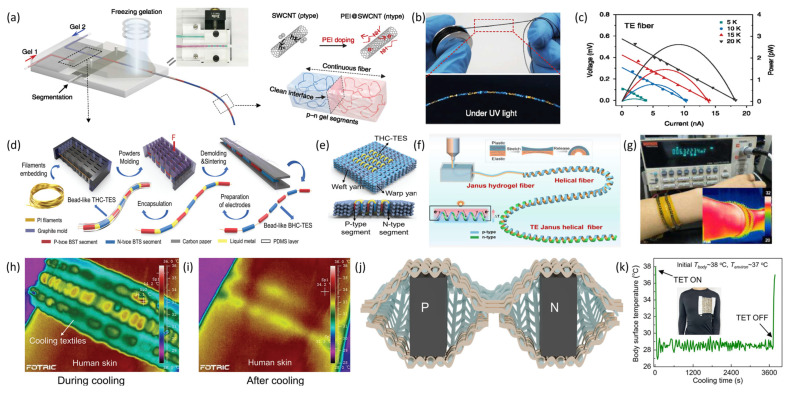
Thermoelectric Power Generation and Thermoelectric Refrigeration Functional Fibers. (**a**) Schematic diagram of digitally controlled continuous alternating extrusion of p/n-type thermoelectric fibers. The hydrogel network effectively confines heterogeneous molecular particles within the matrix, forming alternately arranged p/n-type thermoelectric fibers. (**b**) Photographs of the prepared thermoelectric fibers under natural light (top) and ultraviolet light (bottom). (**c**) Thermoelectric performance of an out-of-plane thermoelectric textile woven from a single fiber comprising 8 p/n thermoelectric pairs, under different temperature differences between the substrate and the environment. (**a**–**c**) reproduced with permission from [169]. distributed under the terms of the Creative Commons Attribution License (CC BY 4.0). (**d**) Schematic diagram of the preparation process for bead-like THC-TES.F represents the pressure exerted by the applied force. (**e**) Schematic diagram of the TET structure (top: overall view; bottom: cross-sectional view, showing the vertical alternating arrangement of p-type and n-type segments). (**d**,**e**) reproduced with permission from [170]. Copyright (2022) RSC. (**f**) Schematic diagram of the design and preparation of ultra-stretchable p/n alternating Janus helical hydrogel fibers. (**g**) Image of Janus helical hydrogel fibers, composed of 90 serially connected p/n units, worn on the human forearm for collecting body heat; the inset shows the temperature gradient distribution of the device. (**f**,**g**) reproduced with permission from [173]. Copyright (2025) American Chemical Society. (**h**,**i**) demonstrate the temperature regulation effect of the TET device on the human skin surface: the skin temperature before cooling was 34.2 ± 1 °C (**h**), and decreased to 32.3 ± 1 °C after cooling (**h**,**i**) reproduced with permission from [170]. Copyright (2022) RSC. (**j**) Schematic diagram of the cross-sectional structure of a TET unit, showing the contribution of each component to the total thermal conductance of the system under a vertical temperature difference. (**k**) Changes in human body temperature before and after TET activation/deactivation in an ambient temperature of 37 ± 1 °C; the inset shows the practical installation of the TET on a T-shirt. (**j**,**k**) reproduced with permission from [174]. distributed under the terms of the Creative Commons Attribution License (CC BY 3.0).

Single-fiber thermoelectric devices typically utilize a periodic electrical series connection of p-type and n-type thermoelectric materials to facilitate electrical-thermal conversion and textile design. In p-type thermoelectric materials, the predominant charge carriers are holes, and the direction of carrier movement aligns with the electric current. Conversely, in n-type thermoelectric materials, electrons are the primary carriers, and their movement opposes that of the electric current. Generally, both organic and inorganic-based p-n segmented thermoelectric fibers or yarns can be fabricated into flexible three-dimensional thermoelectric textiles (TETs). Fiber-based thermoelectric devices mainly serve two applications based on the two thermoelectric effects. The first application involves thermoelectric power generation devices designed for self-powered wearable electronics, operating on the Seebeck effect, which harvest heat from the human body or electronic devices to generate electricity. Ding et al. [169] achieved automated, continuous, alternating extrusion through programming and computer control, thereby fabricating p-n alternating PVA/SWCNT thermoelectric fibers, as depicted in Figure 13a. The n-type material within the fiber was obtained by modifying SWCNTs doped with PEI, as shown in Figure 13b. By freezing the connection points between p-n segments at low temperatures, adjacent gel segments could be bonded together, with the p-n junctions displaying clear interfaces. A thermoelectric fiber composed of eight p-n pairs generated an open-circuit voltage of 0.52 mV under a temperature difference of 20 K, as illustrated in Figure 13c. Zheng et al. [170] constructed a structurally stable thermoelectric (TE) string by sequentially depositing p-type Bi_0.4_Sb_1.3_Te_3_ (BST) and n-type Bi_2_Te_2_Se_0.2_ (BTS) segments onto a polyimide fiber, interconnecting them with liquid metal, and ultimately encapsulating the assembly with a polydimethylsiloxane (PDMS) elastomer, as illustrated in Figure 13d. Utilizing elastic yarn as the warp yarn and a combination of cotton yarn with a ternary hierarchical coaxial TE wire as the weft yarn, they fabricated a three-dimensional multi-layer woven thermoelectric tapestry (TET) (Figure 13e), which delivered an exemplary output power of 0.582 W/m at a ΔT = 25 K. Deng et al. [171] applied a triple treatment involving sulfuric acid (H_2_SO_4_), sodium borohydride (NaBH_4_), and 1-ethyl-3-methylimidazolium dicyanamide (EMIM:DCA) to wet-spun PEDOT:PSS fibers at ambient temperature, resulting in a substantial enhancement of their thermoelectric characteristics. The thermoelectric device (TED) assembled from these fibers achieved an output voltage of (0.49 ± 0.02) mV at a ΔT of 25 K, with a power density reaching (0.28 ± 0.04) μW/cm^2^. Li et al. [172] introduced an innovative approach to fabricating high-performance flexible thermoelectric fibers by preparing PEDOT:PSS-coated tellurium nanowires (PC-TeNWs) via a hydrothermal process, followed by the production of PEDOT:PSS/PC-TeNWs composite thermoelectric fibers through wet spinning. By adjusting the PC-TeNW content, the multicomponent composite fibers attained a power factor (PF) of 385.42 μW/m·K^2^ at room temperature. A four-leg flexible thermoelectric generator (f-TEG) assembled from these composite fibers yielded a maximum power output of 1.456 μW at a ΔT of 37.6 K, corresponding to a power density of 21.382 μW/cm^2^. Wang et al. [173], inspired by plant tendrils, developed a novel superelastic Janus helical hydrogel thermoelectric fiber for harvesting human body heat, as depicted in Figure 13f. Exploiting a biological strain mismatch mechanism, they fabricated Janus helical fibers composed of sodium polyacrylate (PANa) and PANa/single-walled carbon nanotube (PANa-SWCNT) hydrogels with controllable diameters. These fibers maintained stable thermoelectric performance even under a strain of 650%. A wristband equipped with 90 pairs of alternating p/n Janus helical hydrogel fibers was capable of harvesting heat from the human body, producing approximately 15.8 mV at a temperature difference of 37.6 K (ambient temperature of 0 °C), as shown in Figure 13g.

Based on the Peltier effect, thermoelectric cooling devices can be designed to transfer heat from inside a system to the outside. Compared to traditional cooling equipment, thermoelectric cooling devices offer advantages such as small size, no noise, and ease of integration. They hold significant potential in the fields of personal thermal management, electronic device cooling, and medical equipment. Zheng et al. [170] utilized a woven TET as a cooling device; at room temperature, applying an external current of 140 mA to the TET resulted in a temperature drop of 3.1 K on the cold side of the fabric, and this cold-side temperature remained stable for 30 min. In a practical scenario, when a TET-woven wristband was placed on a human arm, a surface temperature reduction of 1.9 K was detected on the arm (Figure 13h,i). Jing et al. [174] reported a simple and scalable composite strategy for thermoelectric fabrics. By directly weaving rigid inorganic TE pillars into flexible textiles, a composite textile with a bilayer structure was constructed (Figure 13j). This method enables the fabrication of large-area (1550 cm^2^), washable, skin-conformable, and truly wearable thermoelectric textiles (Figure 13k). Through the design of a heat sink array on the e-textile, it demonstrated rapid and stable body surface cooling of 11.8 K and a cooling capacity of approximately 553.72 W/m^2^ under a mild breeze environment at 34 °C. Although thermoelectric cooling is a highly promising and environmentally friendly thermal management method, factors such as the intrinsic thermal resistance of the thermoelectric cooling materials, the thermal resistance of the composite fabric materials, and the Joule heat generated during the heat dissipation process can significantly impact the overall cooling efficiency of the device.

Thermoelectric materials facilitate the reciprocal conversion of electrical and thermal energy through various thermoelectric effects, demonstrating significant potential in domains such as personal thermal regulation, electronic device thermal management, and power provision for wearable smart devices, Table 4 provides a concise summary of the materials and performance metrics of thermoelectric fibers. At present, thermoelectric materials are primarily classified into two categories: inorganic materials, exemplified by Te-based thermoelectric substances, and organic materials, exemplified by conductive polymers such as polyaniline (PANI) and polypyrrole (PPy). Among these, inorganic materials display superior thermoelectric performance; however, they are more expensive and less flexible than organic alternatives, rendering them less suitable for fiber processing. Single thermoelectric fibers can be fabricated via methods including thermal drawing, electrospraying, and wet spinning. Currently, the thermoelectric efficacy of devices based on such fibers is generally markedly lower than that of thin-film thermoelectric devices, and the mechanisms to enhance thermoelectric performance remain inadequately developed. Furthermore, for composite thermoelectric fibers, achieving a balance between thermoelectric properties and stretchability constitutes a significant challenge. Ideally, thermoelectric fiber devices should feature characteristics such as high integration, low cost, multifunctionality, safety, and durability. An additional pertinent issue involves the development of measurement methodologies for fiber thermoelectric properties. Presently, parameters such as the Seebeck coefficient, electrical conductivity, and thermal conductivity are measured separately across multiple samples, with sample variability potentially leading to error propagation and substantial inaccuracies in the thermoelectric figure of merit (*ZT*). Future directions include the adoption of integrated in situ measurement techniques—such as the 3ω-T type, T-type, and suspended device methods—that enable multi-parameter assessment within a single sample. Concerning future developments, the primary objective is to further investigate the fundamental mechanisms underlying the enhancement of energy conversion efficiency in fiber-based thermoelectric devices and to increase their output power. Additionally, strategies for low-cost and high-efficiency production of thermoelectric fibers merit exploration. For applications in personal thermal management, improvements in the flexibility, breathability, and comfort of thermoelectric fibers for wearable use are essential. As power sources for wearable electronic devices, systematic development of wearable energy supply systems based on thermoelectric fibers is imperative to augment overall endurance and stability.

### 4.2. Joule-Heated Fiber

In addition to the thermoelectric effect, the Joule heating effect also facilitates the conversion of electrical energy into thermal energy [182]. The Joule heating effect describes the phenomenon whereby an electric field, generated by a voltage difference across a solid or liquid with finite electrical conductivity, drives a flow of charge carriers, typically electrons. The directional movement of these carriers induces thermal motion through collisions with ions within the conductive material. For a purely resistive circuit, the power associated with Joule heating can be expressed as:

(7) $$Q=I^{2}Rt,$$

where *Q* denotes heat, *I* represents current, *R* is resistance, and (*t*) stands for time. Joule electric heaters have advantages like simple structure and easy design and manufacturing. Currently, fiber and textile materials based on the Joule heating effect mainly fall into two groups. The first group includes fibers made directly from intrinsically conductive materials, such as metal wires [183], carbon materials [184], and conductive polymers [185]. The second group involves composite fibers with extrinsic conductivity, created by embedding conductive materials (e.g., metal particles, metal nanowires, carbon materials) into other fiber matrices through methods like coating [186], wet spinning [187], 3D printing [188], and in situ growth [189]. Joule-heating fibers offer the benefits of high flexibility, lightweight, and efficient heating, making them widely useful in personal thermal management in extreme conditions, healthcare, and military applications.

**Figure 14:**
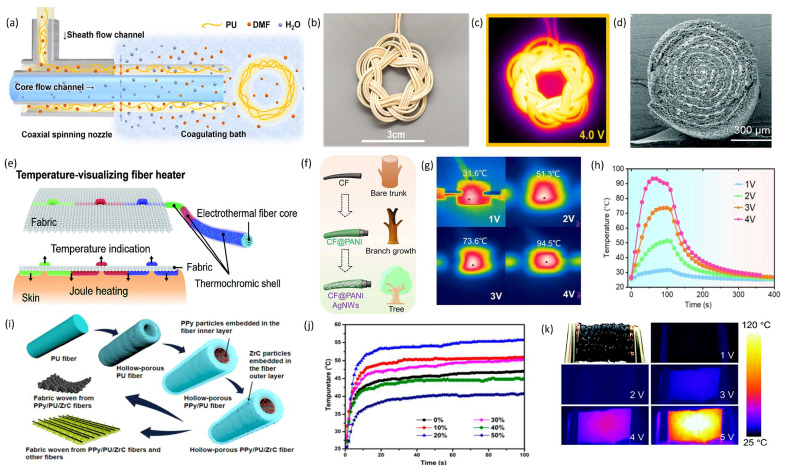
Joule Heating Fibers. (**a**) Schematic diagram of coaxial wet spinning preparation, showing the gradual solidification of PHF through the exchange of DMF and H_2_O. (**b**) Physical photograph of a woven flexible flower-shaped fiber heater. (**c**) Infrared thermal image of the woven flexible heater operating at 4.0 V. (**a**–**c**) reproduced with permission from [190]. Copyright (2022) Elsevier. (**d**) Scanning electron microscope image of the superstructure fiber cross-section. (**e**) Schematic diagram of the structure and working principle of a wearable heater with temperature visualization. (**d**,**e**) reproduced with permission from [191]. Copyright (2022) Wiley. (**f**) Schematic diagram of the PAg_3_ branch-trunk interlocking structure. (**g**) Surface temperature changes of PAg_3_ under different input voltages and (**h**) the corresponding infrared thermal imaging diagram. (**f**–**h**) reproduced with permission from [189]. distributed under the terms of the Creative Commons Attribution License (CC BY 4.0). (**i**) Appearance and structural schematic diagram of hollow-structured PU/PPy/ZrC fibers. (**j**) Temperature profiles of PPZrF-3 over time under different voltages. (**i**,**j**) reproduced with permission from [195]. Copyright (2022) American Chemical Society. (**k**) Schematic diagram of temperature testing for PTFE/CNT fabric under different voltages and the corresponding infrared thermal images of the fabric under various voltages. reproduced with permission from [188]. Copyright (2024) Wiley.

The utilization of metallic wires as elements for electrical heating embedded within traditional textiles has its origins in the previous century. Composite fibers constructed with metallic coatings present enhanced practicality compared to standalone metal wires. Qi et al. [190] documented the development of a reversibly super-stretchable conductive fiber (LM@PHF). Through the application of a coaxial electrospinning process, fibers with a “core–shell” architecture were produced continuously, as depicted in Figure 14a. The core comprises liquid metal (Galinstan, with a mass ratio of Ga:In:Sn = 62.5:25:12.5%), whilst the shell consists of polyurethane (PU). Modifying the spinning parameters enables precise control over the internal configuration of the hollow fiber. An increase in the inner diameter correlates with a reduction in the electrical resistance of the fiber, thereby enhancing its electrothermal performance incrementally. A flower-shaped electric heater fabricated from these fibers (Figure 14b) was energized under bias voltages between 2.0 V and 4.0 V, attaining steady-state temperatures ranging from 46.12 °C to 116.7 °C. Remarkably, stable and sustained low-power heating was also demonstrated in underwater conditions (Figure 14c). The flexible composite fiber, which encapsulates liquid metal within polyurethane, introduces new avenues for the development of underwater wearable devices. Yu et al. [191] proposed a high-performance, superstructured fiber characterized by high conductivity and consistent electrical properties, based on a liquid metal–microfiber interlocking structure. Utilizing a surface scraping technique, semi-fluidic eutectic gallium-indium liquid metal combined with copper particles (Cu-EGaIn) was partially embedded into an electrospun porous fiber mat. A specific winding process was subsequently used to convert the microfiber membrane into a self-supporting, self-encapsulated superstructured fiber (Figure 14d). The numerous Cu-EGaIn convex structures within the fiber flatten when stretched to maintain the effective conductive volume, ensuring consistent electrical performance during deformation. The superstructure fiber demonstrates excellent qualities, such as a high electrical conductivity of 1.5 × 10^6^ S/m, a tensile strain of 629%, and nearly unchanged conductivity under strain. Adding metallic materials gives the fiber enhanced electrothermal performance at low voltages, reaching 94 °C at just 0.9 V with quick thermal response. Based on this, a temperature-visualizable electrothermal fiber heater was created by spraying a mixture of thermochromic materials and a TPU matrix onto the conductive fiber surface(Figure 14e). Besides metallic materials, carbon-based materials are also considered promising electrothermal options due to their good conductivity and processability. Tian et al. [192] developed a dual aerogelization technique to produce carbon aerogel nanofibers with a crimped structure (crimp ratio of 28.3%). Using these fibers, they designed a super-fabric for energy harvesting and thermal management. When applying a voltage of 3–6 V, the fabric temperature increased from 15.1 °C to 48 °C. Furthermore, by adjusting the voltage, the super-fabric could reach a higher temperature of 150 °C within 30 s, demonstrating its quick thermal response and efficient electrothermal conversion, suggesting potential applications in body temperature regulation under extreme environments. Chai et al. [193] developed a conductive composite fiber composed of polytetrafluoroethylene (PTFE) nanofibrils and carbon nanotubes (CNTs) utilizing an innovative in situ fibrillation technique (see Figure 14k). The composite fiber demonstrates high and stable electrical conductivity, measured at 185 S^−^/m. The network constituted by CNTs within the fiber effectively enhances its electrothermal characteristics. Textiles fabricated from this fiber can be heated to temperatures exceeding 130 °C under a low external voltage (<5 V) and sustain this temperature over an extended period. Additionally, the fiber exhibits excellent chemical resistance and high mechanical strength, rendering it a promising candidate for protective clothing in extreme environments. Zhou et al. [194] designed a multifunctional composite fiber featuring a′ branch-trunk″ interlocked micro/nanostructure based on a hierarchical′ three-in-one″ multi-scale design (see Figure 14f). This fiber comprises three one-dimensional fibrous materials: carbon fiber (CF), polyaniline (PANI), and silver nanowires (AgNWs). The composite synergistically combines the excellent flexibility of CF, the robustness of PANI, and the superior conductivity of AgNWs. The integration of these one-dimensional materials and the porous multi-fiber architecture establish efficient internal thermal conduction pathways, enabling the heating device to exhibit superior Joule heating performance under low voltage conditions (see Figure 14g,h). Conductive polymers possess significant potential in medical and textile applications owing to their intrinsic high flexibility, tunable conductivity, and ease of processing. Commonly used conductive polymers include polyaniline, polypyrrole (PPy), and poly(3,4-ethylenedioxythiophene) (PEDOT). PPy is synthesized via pyrrole oxidation or electrochemical polymerization and demonstrates high electrical conductivity (10^4^ S/cm) in its undoped state. Its low cost and relatively straightforward fabrication processes make it a widely employed conductive polymer in wearable heating devices. Yan et al. [195] developed a hollow porous composite fiber, illustrated in Figure 14i, which incorporates PPy and zirconium carbide (ZrC) particles into the inner and outer layers of a polyurethane (PU) fiber, respectively (see Figure 8f,g). The effects of varying ZrC content and different PPy/PU ratios on the mechanical, thermal, and electrical properties of the composite fiber were investigated. Following comprehensive evaluation, PPZrF-3 (containing approximately 3 wt% ZrC) was selected for subsequent stretching and washing tests. The heating performance of PPZrF-3 under a low applied voltage 0.5–2 V) ranged from 34.9 °C to 55.8 °C. The surface temperature of the washed and stretched samples initially increased, then decreased, but all samples demonstrated satisfactory heating performance (see Figure 14j).

In summary, electrothermal textiles have gained significant attention in recent years for their applications in thermal management in extreme environments, military uses, and medical fields. Significant progress has also been made in electrothermal composite fibers. However, large-scale use of electrothermal fibers still faces many challenges. First, designing and manufacturing composite fibers often involve complex processes and strict conditions, which increase the costs of related products. Regarding materials, because single materials have poor stability and obvious limitations, most current electrothermal fibers are made using composite materials. Yet, issues with durability, interactions between different materials, and service life remain unresolved. Second, the comfort provided by electrothermal fiber fabrics is generally not optimal. Finally, integrating power sources into electrothermal fibers raises safety concerns, and these fibers often experience high heat loss and low thermal efficiency.

### 4.3. Photothermal Fibers

Solar energy constitutes the primary source of nearly all energy on Earth. At present, two principal methods are employed for the direct utilization of solar energy: photovoltaic power generation and solar thermal storage. Photothermal conversion, which permits the direct transformation of light energy into thermal energy for practical application, has attracted considerable scholarly and industrial interest within the domain of solar thermal technologies. Solar radiation reaches the Earth in the form of ultraviolet, visible, and infrared light [196]. Photons with different energy levels have unique vibrational frequencies, and when they interact with matter, they can raise the object’s temperature. Photothermal materials are those that can transform sunlight into heat via the photothermal effect. They find wide use in various areas such as biomedicine [197], energy [198], environmental management [199], and seawater desalination [200].

Based on how electrons or bandgap structures in photothermal materials interact with electromagnetic radiation, the mechanisms for achieving photothermal conversion can be categorized into: plasmonic local heating, non-radiative relaxation in semiconductors, and thermal vibrations within molecules. Depending on the photothermal mechanism, these materials can be further classified into plasmonic metals, semiconductors, carbon-based materials, and polymeric materials. The process of photothermal conversion involves two stages: the absorption of light energy and its transformation into heat. Therefore, the efficiency of this conversion mainly depends on the material’s ability to absorb light and turn it into heat [201]. The light absorption efficiency is primarily determined by the material’s composition and physical structure. By considering intrinsic properties such as reflectivity, absorptivity, and transmissivity, composite materials with improved light absorption can be strategically designed [202]. Common strategies to enhance light absorption include plasmonic metal modification [203], defect engineering [204], and the development of hybrid materials [205]. When it comes to surface structure, creating a three-dimensional light-trapping space on the material’s surface—allowing sunlight to enter and reflect multiple times—can be achieved through surface morphology design. Typical structural designs include microporous structures [206], hierarchical structures [207], and biomimetic microstructures [208].

Photothermal fibers are fiber devices endowed with photothermal conversion capabilities, achieved through the integration of photothermal materials into fibers or conventional textiles via techniques such as electrospinning, wet spinning, sol–gel processes, and coating methods. These fibers possess the ability to directly transmute light energy into thermal energy, thereby facilitating their utilization in fields including personal thermal regulation, wearable optoelectronic devices, medical textiles, seawater desalination, and solar steam generation. The subsequent sections will provide a review of recent advancements in the field of photothermal fibers, categorized according to various types of photothermal materials.

**Figure 15:**
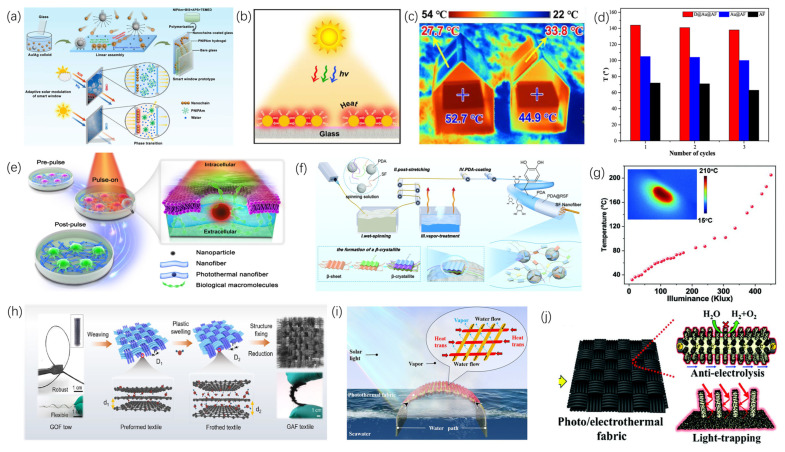
Photothermal functional fibers. (**a**) Schematic of an adaptive solar-regulating smart window fabricated using thermal plasma gold nanochains and thermochromic hydrogels. (**b**) Illustration of the photothermal conversion of in-plane one-dimensional gold nanochains. (**c**) Infrared thermal images (bottom) of two acrylic model houses installed with ordinary glass windows and smart windows, respectively, in an outdoor environment. (**a**–**c**) reproduced with permission from [210]. Copyright (2021) American Chemical Society. (**d**) Photothermal temperatures of D@Au@AF, Au@AF, and AF over three experimental trials. reproduced with permission from [212]. Copyright (2021) Elsevier (**e**) Schematic diagram of the mechanism by which thermal nanofibers achieve intracellular delivery via membrane permeabilization. reproduced with permission from [211]. Copyright (2021) Springer Nature. (**f**) Illustration of polydopamine nanoparticle (PDANP)-functionalized regenerated silk fibroin (RSF) fibers. (**g**) Temperature evolution of the fiber membrane under different light intensities. (**f**,**g**) reproduced with permission from [217]. Copyright (2022) Wiley (**h**) Schematic of the preparation process for graphene oxide fiber (GAF) fabric and photographs of the actual fabric. reproduced with permission from [216]. Copyright (2023) Springer Nature. (**i**) Schematic diagram of the structure of an ABPF interfacial solar evaporator. reproduced with permission from [219]. Copyright (2024) Springer Nature. (**j**) Schematic of a photothermal–electrothermal fabric combining an array-induced light-trapping effect with a core–shell structure-induced capacity for resistance to electrolyte penetration. Reproduced with permission from [220]. Copyright (2025) American Chemical Society.

Precious metal materials, such as gold and silver, are classical plasmonic photothermal materials. The photothermal effect exhibited by metallic nanostructures results from the localized surface plasmon resonance (LSPR) phenomenon. When the frequency of incident light coincides with the natural frequency of electrons on the metal surface, photons induce coherent oscillation and redistribution of electrons [201]. Upon absorption of incident photon energy, plasmonic nanostructures on the metal surface undergo electron transitions, thereby generating hot electrons [209]. Electron-electron scattering subsequently causes a localized temperature increase within the metal, which is then transferred to the surrounding medium via electron-phonon and phonon-phonon scattering mechanisms [210]. Guo et al. [211] integrated one-dimensional gold nanochains with thermochromic hydrogels to develop an intelligent window for solar regulation (see Figure 15a). In this configuration, metal nanowires serve as photothermal materials that absorb solar energy and convert it into heat, functioning as thermal switches for the thermochromic hydrogel (see Figure 15b). By modulating electrostatic interactions among nanoparticles during planar linear assembly, the gold nanochains acquired broadband light absorption properties. As illustrated in Figure 15c, the smart window based on one-dimensional gold nanochains blocks 94.1% of solar radiation within the 300–2500 nm wavelength range while transmitting 71.2% of visible light prior to optical switching, thereby maintaining visual comfort indoors. Jiang et al. [212] reported a photothermal composite fiber featuring a three-layer coaxial structure (D@Au@AF). A nano-gold layer and a polydopamine-based photothermal coating were self-assembled onto the surface of plasma-treated aramid fibers, constructing a composite photothermal fiber with a three-layer architecture (see Figure 15d). The synergistic photothermal effect between the nanometallic layer and the polydopamine layer enhances the photothermal conversion efficiency of the composite fiber. Under irradiation with 275 W infrared light, the maximum temperature of D@Au@AF reached 144 °C.

Compared to metallic materials, semiconductor materials provide advantages such as lower cost, ease of synthesis, and low toxicity. The photothermal effect of semiconductor materials relies on non-radiative relaxation phenomena. The fundamental principle of photothermal conversion is that when the incident photon energy is equal to or exceeds the bandgap energy of the material, the material absorbs the photon energy, exciting electrons and generating electron-hole pairs. Upon returning to the band edge, the excited electrons release energy either through radiative relaxation, emitting photons, or via non-radiative relaxation in the form of phonons. During non-radiative relaxation, energy is released as heat [213]. Semiconductor composite photothermal films typically incorporate transition metal oxides and metal sulfides (such as Rb_x_WO_3_, WO_3−x_, TiO_x_, etc.). Cheng et al. [214] combined tungsten oxide nanoparticles, with diameters less than 100 nm and prepared by ball milling, with polyurethane (PU) in dimethylformamide (DMF). After solvent evaporation, a WO_3−x_/PU nanocomposite was produced, demonstrating that the photothermal temperature increased with higher WO_3−x_ concentration and a greater degree of reduction. At a WO_2.72_ concentration of 7 wt%, the temperature rise in the polymer nanocomposite due to photothermal effect reached as high as 120 °C, with a heating rate of approximately 100 °C/min under 150 W infrared irradiation. Xiong et al. [215] introduced a straightforward approach based on photothermal electrospun nanofibers (PEN). Photosensitive iron oxide nanoparticles (IONPs) were embedded within biocompatible nanofibers via electrospinning. Even in the absence of direct contact between cells and the photothermal nanoparticles, a membrane permeability effect comparable to traditional nanoparticle-sensitized photoporation could be achieved, offering a safer alternative for intracellular delivery of biological agents.

The photothermal conversion of carbon-based materials primarily depends on the thermal motion of molecules, where the energy of photons induces the excitation of loosely bound electrons from the π orbital to the π* orbital within the material [201]. When the excited electrons relax back to the ground state, energy is released as heat. Compared to metallic and metal oxide materials, carbon-based materials present advantages in terms of safety and environmental friendliness. Zhao et al. [181] reported an integrated solar heating yarn, manufactured by twisting carbon nanotube fibers with cotton yarns and treating them with carbon black paste, which is primarily utilized for constructing efficient fabric evaporators [106]. This distinctive hybrid yarn structure demonstrates a significant gradient photothermal difference (5 °C) under dry conditions. Qi et al. [216] achieved large-scale fabrication of high-performance graphene aerogel fiber fabrics through a plastic expansion strategy (Figure 15h). The plastic expansion method permits the production of aerogel fibers with high orientation, facilitating the interconnection of the porous network within the aerogel fibers and enabling effective control over parameters such as fiber density and porosity. Due to the spectral absorption properties of graphene, the surface temperature of the resulting fiber fabric increases from 58 °C to 96 °C within 120 s under simulated solar irradiation ranging from 100 W/m^2^ to 200 W/m^2^.

Organic polymers with conjugated structures have recently gained prominence as a novel category of photothermal nanomaterials owing to their versatile molecular frameworks, robust absorption in the near-infrared spectrum, high efficiency in photothermal conversion, and favorable biocompatibility. Analogous to carbon-based photothermal substances, the majority of organic materials produce heat primarily through lattice relaxation processes triggered by the absorption of light energy. Predominantly utilized polymer-based photothermal agents include polypyrrole (PPy), polydopamine (PDA), and polythiophene (PT), among others. As reported by Chen et al. [217], a wet-spinning technique was employed to produce a regenerated silk fibroin (RSF) fiber solution embedded with polydopamine (PDA) nanoparticles, resulting in PDA@RSF fibers. These fibers were subsequently coated with PDA through oxidative self-polymerization of dopamine, forming composite photothermal fibers (see Figure 15f). The PDA coating on the PRP fibers imparted the ability to efficiently absorb near-infrared and solar radiation. Textiles fabricated from PRP fibers demonstrated outstanding photothermal conversion capabilities, with temperatures rising from ambient conditions to above 200 °C upon illumination (see Figure 15g). Li et al. [218] designed a core–shell structured nanophotothermal fiber using coaxial electrospinning, with an olive oil solution of AIE-active molecules constituting the core and PVDF-HFP forming the shell. The integration of AIE-active molecules within the core–shell nanofiber architecture maximized molecular motion retention and minimized radiative decay. Photothermal investigations demonstrated that the fabricated core–shell nanofibers attained a photothermal conversion efficiency of up to 22.36%, which is twenty-six times greater than that of non-core–shell nanofibers. Yang et al. [219] introduced a novel, scalable, and recyclable high-efficiency arch-bridge structured photothermal fabric solar evaporator (see Figure 15i). Utilizing traditional shuttle weaving technology, efficient warp-direction water channels were constructed, and through the strategic design of weaving patterns and water channel regulation, effective gas–liquid transport pathways were established, thereby enhancing the interfacial evaporation performance of the photothermal fabric. Inspired by the hierarchical array structure found on bird-of-paradise feathers surfaces, Inspired by the hierarchical array structure on the surface on bird-of-paradise feathers, Li et al. [220] created a bio-inspired photothermal/electrothermal CF/TiO_2_/PPy fabric capable of all-weather evaporation (see Figure 15j). This composite fiber, produced through a two-step process of hydrothermal synthesis and in situ polymerization, has a carbon fiber core coated with a TiO_2_/PPy nanorod array. These nanorods effectively trap light, allowing broad-spectrum absorption from 280 to 2500 nm with up to 95.5% efficiency. The fabric made from this fiber achieved an evaporation rate of up to 2.22 kg·m^−2^·h^−1^ under sunlight, offering a new method for high-efficiency seawater desalination in all weather conditions.

Photothermal materials transform radiative energy from various wavelengths of sunlight into thermal energy via the photothermal effect. Common materials exhibiting photothermal conversion include plasmonic materials, primarily noble metals such as gold; semiconductor materials, exemplified by metal oxides; carbon-based materials; and organic polymers with conjugated structures. Photothermal fibers are composite fibers endowed with photothermal conversion capabilities through the integration of nanomaterials into conventional fibers, employing methods such as electrospinning, sol–gel processes, coating techniques, and wet spinning. Currently, these materials are extensively applied in personal thermal management, biomedical applications, seawater desalination, and steam power generation. Nonetheless, several challenges impede the development and design of photothermal materials. Firstly, the efficiency of photothermal conversion is generally limited, primarily due to inadequate light absorption, which can be enhanced through strategic material selection, elemental doping, and surface microstructure engineering. Secondly, improving the stability and durability of these materials remains imperative, achievable via surface engineering techniques, including coating and functionalization, as well as the development of composite materials. Finally, the high cost associated with manufacturing photothermal fiber devices and their constituent materials restricts large-scale production, a predicament that may be alleviated by employing cost-effective alternative materials, such as replacing gold, palladium, and other noble metals with copper, and by adopting scalable synthesis methods to reduce manufacturing expenses.

### 4.4. Thermally Actuated Fibers

In recent years, advances in wearable technology have led to the creation of wearable devices that not only monitor the wearer’s environment and physiological data but also generate feedback and responses to external stimuli, resulting in the development of so-called smart wearable devices [221,222]. Actuators, as specialized energy conversion devices, undergo reversible linear bending and torsional movements due to structural and material property changes when exposed to environmental stimuli, converting received energy into mechanical energy. Based on their actuation mechanisms, actuators can be categorized into rigid and soft types. Soft actuators, in particular, are made of flexible, lightweight, and conformable materials. Compared to traditional mechanical systems, fiber-based materials offer advantages such as simplicity, flexibility, and anisotropy [223]. Additionally, they can be processed into two- or three-dimensional structures through textile manufacturing techniques, showing significant potential in soft robotics and wearable devices [224,225]. Fiber actuators work by sensing external environmental changes—such as light, heat, electricity, and humidity—and generating mechanical motion. They achieve this primarily through three mechanisms: changes in molecular chain alignment, variations in fiber volume, and shifts in the distance between fibers [226,227,228,229]. Depending on the type of external energy driving them, fiber actuators are classified as electrical, thermal, photonic, magnetic, or moisture-driven actuators.

**Figure 16:**
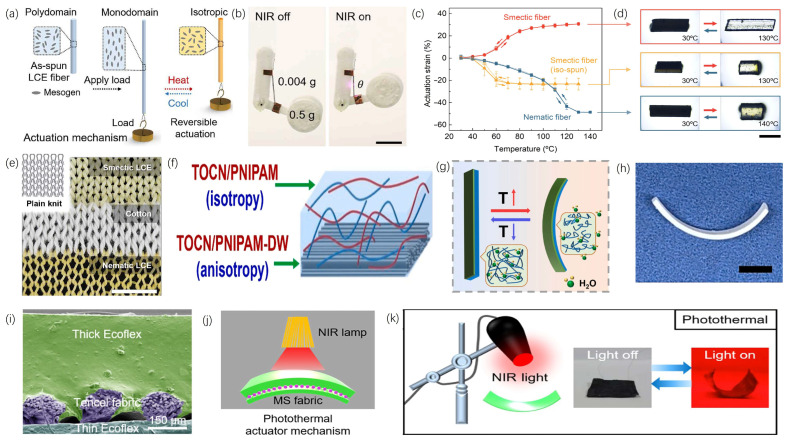
Thermally Actuated Functional Fibers. (**a**) Schematic illustration of the actuation mechanism of LCE fibers. The nascent microfiber exhibits a polydomain state, which transitions to a monodomain state under applied tensile force, aligning the liquid crystal mesogens axially. Upon heating the stretched LCE microfiber, the nematic-to-isotropic phase transition induces axial contraction, with full recovery to the initial length upon cooling. (**b**) Upon near-infrared light irradiation of the fiber bundle, biomimetic bicep contraction causes arm bending. (**a**,**b**) reproduced with permission from [231]. Copyright (2021) AAAS. (**c**) Thermal actuation strain curve of the LCE fiber. (**d**) Corresponding optical micrographs. Scale bar: 500 μm. Actuation strain (%) = [L(T) − L_0_]/L_0_ × 100 (%), where L(T) is the fiber length at temperature T and L_0_ is the initial length. Negative and positive values indicate thermal contraction and thermal elongation behaviors, respectively. (**e**) Photograph and structure of the plain-knitted LCE fabric. Scale bar: 1 cm. (**c**–**e**) reproduced with permission from [232]. Copyright (2025) AAAS. (**f**) Structure of the hydrogel actuator and its thermal-responsive deformation process.The red line represents PNIPAM, the blue line denotes TOCNs, and the green line signifies DW. (**g**) Thermal-responsive deformation process of the hydrogel actuator. (**h**) Photographs of the thermal actuation of the hydrogel actuator with a 60° cutting angle. (**f**–**h**) reproduced with permission from [234]. Copyright (2025) Elsevier. (**i**) SEM image of the MS fabric, comprising three asymmetric layers: a thick Ecoflex layer (green), MXene/AgNPs-modified Tencel fabric (purple), and a thin Ecoflex layer (cyan). (**j**) Photothermal actuation mechanism of the MS fabric. (**k**) Near-infrared photothermal actuation of the MS fabric. (**i**–**k**) reproduced with permission from [235]. Copyright (2022) Springer Nature.

A thermal actuator converts environmental heat into mechanical energy through the material’s detection and response to thermal signals. Typically, thermally actuated fibers induce movement by altering the arrangement of molecular chains within the fiber, changing its volume, or modifying the spacing between fibers. There are three main methods for thermal actuation: the electrothermal effect based on Joule’s law, the near-infrared photothermal effect, and thermal radiation [230]. Shape memory materials are an important type of thermal actuators. He et al. [231] designed and created an LCE microfiber thermal actuator using electrospinning technology. Its working principle is shown in Figure 16a. When the LCE microfiber is heated by the environment or exposed to a near-infrared laser, it can produce an actuation strain greater than 50% in less than one second. Compared to human muscle, the LCE microfiber has similar mechanical properties: actuation strain (>50%), actuation stress (0.3 MPa), response speed (300%/s), and work density (203 kJ/m^3^). Moreover, at high temperatures (90 °C), even after 1,000,000 loading and unloading cycles at a maximum strain of 20%, the LCE microfiber showed no signs of performance degradation. Lee et al. [232] described a thermally elongating LCE fiber produced through ultraviolet-assisted melt spinning. By controlling the liquid crystal phase during the spinning process, it is possible to fabricate LCE fibers with various orientations and actuation directions, typically categorized as either contractile or extensile fibers. The extensile lamellar liquid crystal elastomer fiber presented in this study features mesogens aligned perpendicular to the fiber axis, enabling spontaneous elongation upon heating. Its thermomechanical actuation strain curve and polarized optical micrographs are depicted in Figure 16c,d. Upon exceeding the critical temperature, the smectic-phase fiber undergoes spontaneous elongation of 31%, whereas the nematic-phase fiber contracts thermally by 49%. These findings bear significant implications for the design of smart fibrous textiles (see Figure 16e). An alternative approach to attaining shape memory and reversible actuation involves the construction of materials with differing thermal characteristics into distinctive bilayer structures. Ionov et al. [233] fabricated a dual-sided structural fiber comprising two polymers arranged side-by-side utilizing 3D printing and melt-spinning techniques. Upon cooling, materials such as ABS or PLA undergo contraction responses, causing the entire fiber to bend toward the respective side. Conversely, heating melts the materials, allowing the fiber to revert to its original shape. This bilayer Janus fiber attains shape memory functionality by exploiting the contrasting thermal responses of the constituent materials to cold and hot stimuli. Lu et al. [234] developed an innovative temperature-responsive wood hydrogel actuator that leverages the inherent anisotropic characteristics of wood fibers. This thermal actuator was fabricated through a one-pot synthesis process, comprising two principal components: an isotropic thermo-responsive hydrogel composed of TEMPO-oxidized cellulose nanofibers and poly(*N*-isopropylacrylamide) (TOCN/PNIPAM), and an anisotropic delignified wood (DW) (refer to Figure 16f). As illustrated in Figure 16g, when the temperature exceeds a critical threshold, the hydrophobic groups of the PNIPAM hydrogel dominate, expelling water and inducing a controlled deformation of the actuator (see Figure 16h). The anisotropic nature of DW facilitates programmable thermal deformation through the adjustment of cutting angles, thereby enabling applications in biomimetics and information encoding. Additionally, materials exhibiting photothermal effects, such as carbon nanotubes, MXenes, and silver nanowires, have been incorporated into the design of thermal actuators. Li et al. [235] reported a multifunctional, integrated smart Tencel fabric that responds to light and electricity. The fabric, called MS fabric, was made by coating Tencel fibers with Ti_3_C_2_T_x_ MXene nanosheets and silver nanoparticles (AgNPs). The MS fabric shows excellent ability to convert light and electrical energy into heat, enabling actuation through optical or electrical stimulation. It has a thermally symmetric three-layer structure (Figure 16i), where the middle Tencel layer absorbs near-infrared (NIR) light and turns it into heat, the bottom thick Ecoflex layer acts as the actuation layer because of its high thermal expansion, and the top thin Ecoflex layer serves as an encapsulation. As shown in Figure 16k, the bending angle of the MS fabric increases with temperature, reaching a maximum of 149° at 31 s, then decreases during cooling to room temperature. This demonstrates the quick photothermal actuation ability of the smart wearable device. Recently, Yang et al. [236] developed a simple, solvent evaporation-assisted template method to create liquid crystal elastomer hollow fibers (LCEHFs) with aligned structures along their axis. By combining the effects of thermally induced phase change and mechanically induced orientation change, the LCEHFs can contract about 50% under both thermal and pneumatic stimuli, exceeding the 42% contraction by thermal stimulation alone or the 27% by pneumatic stimulation. Moreover, this method improves the response and recovery speeds of the LCEHFs by up to 300 and 3700 times, respectively, compared to pneumatic actuation. The reinforcement effect also lowers the actuation temperature well below the phase transition temperature, giving the LCEHFs high mechanical performance during actuation.

The variation in volume consequently facilitates the conversion of thermal energy into mechanical energy. Furthermore, the implementation of thermal actuation often requires an accompanying heat absorption process, which can be achieved through electrical heating or photothermal absorption mechanisms. This indicates that thermal actuation fibers are fundamentally a type of composite functional fiber material capable of thermal conversion. Commonly utilized thermal actuation materials include shape memory materials (such as PLA, LCE, etc.), carbon nanotubes, MXenes, and silver nanowires. Thermal actuation fibers can be synthesized via methods such as 3D printing, melt spinning, electrospinning, and surface coating. They are primarily employed in smart textiles, soft robotics, and healthcare sectors, with their performance largely dependent on actuation strain and response speed under thermal stimuli. Currently, thermal actuation fibers face limitations due to high raw material costs and their relatively complex fabrication processes, which hinder scalable manufacturing. Additionally, the durability and stability of fiber-based devices tend to decline significantly during thermal cycling. To overcome these challenges, future research directions for thermal actuation fibers emphasize two key aspects: firstly, the exploration of novel high-performance, low-cost thermal actuation materials, such as biomass-derived materials; secondly, functional integration, whereby the combination of thermal actuation fibers with heat-absorbing materials, energy storage devices, and other components into an integrated system can facilitate more stable and sustainable practical functions.

## 5. Conclusions and Outlook

This paper offers a succinct review of recent developments in functional fibers designed for thermal regulation. Depending on their reliance on external energy input, thermal regulation methodologies are classified into passive and active strategies. Passive thermal regulation primarily facilitates thermal management through intentionally engineered heat transfer and storage processes. Concerning heat transfer, the paper systematically reviews key strategies and recent research advances aimed at enhancing or suppressing heat exchange, from the perspectives of thermal conduction and radiation. This section emphasizes design principles and fabrication techniques for high-thermal-conductivity fibers, thermal insulating fibers, and radiative functional fibers. Regarding heat storage, the design concepts of phase-change thermal storage fibers and their typical applications are summarized, along with prospects for future development. Active thermal regulation involves energy conversion processes between thermal energy and other energy forms. This section investigates thermoelectric fibers based on the thermoelectric effect, electrothermal fibers utilizing Joule heating, photothermal fibers leveraging the photothermal effect, and thermally actuated fibers derived from thermo-responsive mechanisms. Beyond fiber fabrication, the functional applications of these fibers and their corresponding device designs are highlighted, offering broader possibilities for the development and application of thermal management fibers and related devices.

Regarding the thermal functionalization of fibers, although researchers have developed various functional fibers with diverse compositions and structures from the perspectives of thermal transport, storage, and conversion, their design and fabrication strategies generally follow several common developmental trends:

First, there is an emphasis on the intrinsic properties of fibers. Currently, most thermally functional fibers still primarily depend on the inherent thermal properties of the materials themselves, with ongoing research focused on screening and constructing systems with superior intrinsic thermal conductivity, insulation, or energy conversion performance.

Second, there is refined control over the microstructure of fibers. A growing body of work shows that the internal and surface microstructures of fibers significantly influence thermal behavior: constructing ordered thermal conduction pathways within fibers through orientation engineering enhances thermal conductivity, creating porous or hollow structures via coaxial electrospinning or ice-templating methods improves insulation, and regulating interfacial structures through surface coatings or molecular design enables radiative regulation or photothermal responses.

Third, the rise in multifunctional composite fibers responds to complex and changing application scenarios. Single thermal functionalities often fall short of meeting demands, so researchers integrate materials with diverse thermal characteristics into a single fiber using 3D printing, surface coating, and various composite spinning techniques, achieving the coupling of multiple functions such as thermal conduction, thermal storage, photothermal conversion, and thermoelectricity.

Finally, there is a focus on natural and biomimetic materials. Natural materials are low-cost and widely available; for example, lignocellulose is a key raw material for cellulose aerogel fibers, and silk is commonly used as a matrix for multifunctional composite fibers. Meanwhile, biomimetic templates such as the hollow structure of polar bear hair or the micro–nano photonic structures on the cuticle of Saharan silver ants have inspired the design of high-performance artificial fibers. Thermally functional fibers based on natural and biomimetic concepts are emerging as a promising new direction worthy of focused development.

Although significant progress has been achieved in various thermal-regulating functional fibers, there remains considerable scope for enhancement in their design, production, and functional expansion. Based on existing research, additional considerations can be proposed in the following areas.

First, regarding preparation and scalability, currently most functional fibers still face challenges such as complex processes, low yield, high costs, and difficulties in large-scale production, which constitute the primary obstacles to their transition from laboratory development to commercialization. Simplifying processes and reducing costs can be approached from two perspectives: (1) selecting readily available, low-cost raw materials and appropriately modifying natural fibers; (2) optimizing existing preparation routes by introducing continuous production techniques (e.g., thermal drawing, continuous coating, etc.) to enhance efficiency and ensure batch-to-batch consistency.

Second, concerning functional expansion, many thermal-regulating fibers in contemporary research are often designed for single working conditions and exhibit only a solitary function. In complex application scenarios, fibers with a single function frequently fail to deliver optimal overall thermal management performance. Accordingly, developing composite functional fibers that integrate multiple thermal-regulating mechanisms will be a crucial direction. For instance, by coating or blending, a radiative regulation layer can be combined with phase-change heat-storage fibers to construct an integrated thermal regulation system characterized by “absorption–storage–release”.

Third, pertaining to personal thermal management applications, thermal-regulating functional fibers intended for wearable use must ultimately serve as textiles. Beyond thermal regulation performance itself, practical requirements such as comfort, breathability, flexibility, durability, and esthetics must also be considered. Therefore, when designing personal thermal management fibers and fabrics, a balance must be achieved between thermal functionality and wearability to realize more comprehensive practical attributes.

Fourth, regarding testing methods, currently there is a lack of mature commercial testing techniques for key parameters such as thermal conductivity and thermoelectric performance of single or microscale fibers. Existing methods—including photothermal reflection, T-type, 3ω, suspended microdevice, and TDTR—demand high standards of sample preparation and involve complex testing procedures. In thermoelectric performance characterization, stepwise measurements across multiple samples can introduce errors. A more promising approach involves developing in situ, integrated multi-parameter measurement techniques for the same sample to obtain more accurate and comparable performance data.

Fifth, concerning evaluation standards, presently, significant disparities exist in testing environments, instruments, and characterization methods for thermal conductivity, electrical conductivity, and device performance across different material systems, complicating cross-comparison efforts. There is an urgent need to establish relatively unified testing procedures and evaluation index systems, standardizing measurement conditions and error margins for key performance parameters. Concurrently, unified industry standards and scalable manufacturing specifications should be developed to facilitate the industrial application of fiber materials.

Sixth, regarding environmental impact and sustainability, currently the preparation of many functional fibers continues to involve lengthy processes, high energy consumption, and the generation of substantial wastewater and solid waste, raising environmental concerns. Future efforts should focus on both raw materials and processes: on the material side, increasing the use of natural or renewable substrates combined with functional design; on the process side, developing simpler, more efficient, and low-pollution production methods to balance performance improvements with environmental sustainability.

## Figures and Tables

**Table 1:** High Thermal Conductivity Functional Fibers.

Fibers	Fiber Materials	Diameter (μm)	Thermal Conductivity (W·m^−1^·K^−1^)	Preparation Method	Testing Method	Reference
Carbon Fiber	K1100	-	1100	-	-	[33]
Carbon nanotube fibers	Carbon Nanotubes	-	635	Liquid Crystal Spinning Method	-	[28]
Graphene fibers	Graphene	500	1590	Multi-shear flow-assisted wet spinning (MSW)	the steady self-heating method	[53]
Pure boron nitride fibers	Boron Nitride	10	54	polymetric-derived-ceramic	big-MEMS	[41]
Ti_3_C_2_T_x_ fibers	Borate, Ti_3_C_2_T_x_	23	13	Wet Spinning Method	cross-wire geometry method	[42]
BN@XG fibers	Boron nitride nanosheets, xanthan gum	-	8.26	Freeze-drying method	-	[51]
ANF/BNNSs Coaxial Fibers	Boron nitride nanosheets	-	17.2	Coaxial electrospinning method	transient electro-thermal (TET) technique	[43]
TPU fibers coated with EcoFex–BNNSs layer.	TPU, Ecoflex-BNNSs	-	0.844	Electrospinning, Surface Coating	thermal bridge method	[52]
MPGFs	Graphene	15	1070	Melt Spinning Method	steady-state short-hot-wire method	[54]
(PP@G) fibers	PP, Graphene	20	87	Electrostatic Self-Assembly	laser flash method	[59]
Polyethylene Nanofibers	Polyethylene		104–108	Mechanical Stretch		[56]
PE Nanofiber	PE	10–100 nm	90	Localized heating, stretching	Suspended Device Method	[57]
Polyethylene nanofibers	Polyethylene	50 nm	9.3	Electrospinning	Suspended Device Method	[58]

(Abbreviations: Ti_3_C_2_T_x,_ a two-dimensional titanium carbide, where T_x_ represents surface terminations such as –O, –OH, and –F; BN, Boron nitride; XG, Xanthan gum; ANF, Aramid nanofiber; BNNSs, Boron nitride nanosheets; MPGFs, melamine–polyimide gradient foams; PP, polypropylene; G, Graphene; PE, polyethylene).

**Table 2:** Thermally Insulating Functional Fibers.

Fibers	Fiber Materials	Diameter (μm)	Thermal Conductivity (W·m^−1^·K^−1^)	Preparation Method	Testing Method	Reference
Silica aerogel fibers	Silicon dioxide	50	-	Sol–gel	-	[68]
Hollow silica aerogel fibers (SAFs)	Silicon dioxide	130	-	Wet Reaction Spinning Method	-	[72]
Highly transparent silica aerogel fibers	Silicon dioxide	380	0.018–0.023	Reactive Spinning	Experimental Comparative Estimation	[73]
PI Aerogel Fibers	PI	650	28.7 ± 2.0	Freeze spinning	Hot Disk	[75]
NKLC Aerogel Fibers	NKLC	120	0.037	Liquid Crystal Spinning, Freeze Drying	Hot and Cold Plate Test	[76]
All-cellulose fractionated sponge aerogel fibers (CGF)	Cellulose	48	0.023	Flow-assisted dynamic dual-crosslinking strategy	a setup including the transient plane sensor (TPS)	[78]
CA/PAA@CNF Aerogel Fibers	(CNF), (CA/PAA)	430–545	0.054	Distributed Coagulation Coaxial Wet Spinning and Freeze Drying	Hot Disk	[80]
Encapsulated Aerogel Fibers (EAF)	TPU, EAF	500–600	-	Freeze spinning, surface coating	-	[82]
(Y_0.2_La_0.2_Er_0.2_Ho_0.2_Tm_0.2_)_6_MoO_12_ Ceramic nanofibers	(Y_0.2_La_0.2_Er_0.2_Ho_0.2_Tm_0.2_) _6_MoO_12_	0.2–0.3	0.0689	Electrospinning	Hot Disk	[74]
CNFs/PANI Composite Fiber Aerogel	CNF, Polyaniline	1.1–1.9	0.104	Freeze-drying electrospinning, in situ growth	DRE-III-X Thermal Conductivity Tester	[77]
GAFs	ANFs		0.0228	continuous microfluidic spinning technology	thermal conductivity analyzer	[79]

(Abbreviations: SAFs, silica aerogel fibers; PI, polyimide; NKLC, Nanoscale Kevlar Liquid Crystal; CGF, Cellulose Sponge-Aerogel Fibers; CA, Cellulose acetate; PAA, Polyacrylic acid; CNF, cellulose nanofiber; EAF, Encapsulated Aerogel Fibers; TPU, Thermoplastic polyurethane; PANI, Polyaniline; GAFs, gradient all-nanostructure aramid aerogel fibers).

**Table 3:** Phase Change Thermal Energy Storage Functional Fibers.

Fibers	Thermal Storage Material	Preparation Method	Enthalpy of Phase Transition (J/g)	Melting Point (°C)	Freezing Point (°C)	Reference
MTPCM	Lauric acid (LA)	Vacuum Impregnation Method	165.6	41.3	39.3	[120]
AgNW/MXene@Aramid aerogel nanofibers	Polyethylene glycol	Impregnation method	185	-	-	[121]
Wool fibers impregnated with polyethylene glycol	Polyethylene glycol	Impregnation method	-	-	-	[122]
PMMA/SiO_2_ PCM MCs	Paraffin, butyl stearate	Coating Method	19.03/10.1	36.2	30.03	[126]
PW/PVA PCM fibers	RT27 Paraffin (PW)	Microencapsulation Wet Spinning Method	42.67	27	27	[127]
Phase change microcapsules (PCMC)	PVB/PCMC-60	Electrospinning	92.6	32	28	[128]
PEG/PVA PCF	PEG	Electrospinning	72.3	24.6	18.4	[129]
PW@H-KAF	Paraffin	Wet Spinning Method	135.1–172	58	44	[130]
PAN/PEG/SiC PCM fibers	PEG	Centrifugal Spinning	69.91	51.31	33.71	[131]
PEG@TPU/BNNS-es	PEG	Coaxial electrospinning	101	61	36.8	[132]
C18@TEOS/PHBV fibers	n-Octadecane	Coaxial electrospinning	88.3	28.8	23.0	[134]
ScPEG-coated A-GFF	ScPEG	Scalable Spray Drying Method	62.9	55.3	38.8	[135]
PMFs	PEG	Wet Spinning Method	91	26–46	8–38	[136]
PVB@RT 25 GPCF	RT 25	Microfluidic Spinning	-	-	-	[141]
PAN@PW	Octadecane	Coaxial electrospinning	171.	28.8	27	[133]
GC@PEG/PAN	PEG	Electrospinning	74.51	56.61	41.14	[142]

(Abbreviations: MTPCM, Microencapsulated Thermal Phase Change Material; PMMA, Polymethyl methacrylate; PCM, Phase Change Material; PW, Paraffin wax; PVA, Poly(vinyl alcohol); PEG, Poly(ethylene glycol); PVA, polyvinyl alcohol; PCF, Phase change fiber; H-KAF; PAN, Polyacrylonitrile; TPU, Thermoplastic polyurethane; BNNS, Boron nitride nanosheets; TEOS, Tetraethyl orthosilicate; PHBV, Poly(3-hydroxybutyrate-co-3-hydroxyvalerate); PMFs, poly(ethylene glycol)/4,4′-methylenebis(cyclohexyl isocyanate) fibers; PVB, Poly(vinyl butyral); GPCF, Graphene-coated phase change fiber; A-GFF, AgNW-modified AgNW-modified.).

**Table 4:** Thermoelectric Functional Fibers.

Fibers	Preparation Method	Temperature	Seebeck Coefficient (μVK^−1^)	Electrical Conductivity (Sm^−1^)	Thermal Conductivity (W·m^−1^·K^−1^)	Power Factor (μWm^−1^K^−2^)	*ZT*	Reference
Bi_2_Se_3_ core fibers	thermal drawing technology	300 K	−150.85	31,900	1.25	725.9	0.18	[175]
Bi_2_Se_3_ nanowire	thermal drawing technology	Room temperature	−51	150,767	2.05	393.2	0.06	[176]
Bi_2_Se_3_ fiber	thermal drawing technology	300 K	-	-	0.52	1320	0.76	[157]
Single-crystal SnSe core fibers	thermal drawing technology	862 K	~310	5500	0.22	528.55	1.94	[158]
Ag_2_S fiber	thermal drawing technology	Room temperature	−7.7 × 10^−6^	5.9 × 10^4^	2.34	-	4.4 × 10^−4^	[159]
PbTe Nanowires	hydrothermal process	Room temperature	307	273	-	25.73	-	[177]
CNF fibers	wet-spinning approach		56.5	1353	-	432		[160]
carbon nanotube threads	pyrolytic synthesis (eDIPS) method	Room temperature	48	4800	-	11.06	-	[178]
graphene fibers	wet-spinning approach	290 K	−3.9	118,000	137	1.79	3.7 × 10^−6^	[179]
PEDOT: PSS fibers	wet-spinning approach	-	19	8300	-	30	-	[180]
PEDOT: PSS/SWCNT fibers	gelation process and ethylene	Room temperature	17.23	24,330	-	7.23	-	[181]
PC-Ag_2_Te NWs fibers	Hydrothermal reaction, wet spinning method	Room temperature	−61.3	17,370	1.05	65.3	∼0.02	[168]
(PEDOT:PSS/SWCNT@PANI) fibers	wet-spinning approach	Room temperature	43.5 ± 0.7	247,200 ± 2330	-	467.8 ± 10.5	-	[166]

(Abbreviations: Bi_2_Se_3_, Bismuth selenide; SnSe, Tin selenide; PbTe, lead telluride; Ag_2_S, Silver sulfide; CNF, Carbon nanofiber; PEDOT: PSS, poly(3,4-ethylenedioxythiophene):poly(styrenesulfonate); SWCNT, Single-walled carbon nanotubes; PC-Ag_2_Te NWs, poly(3,4-ethylenedioxythiophene):poly(styrenesulfonate) (PEDOT:PSS)-coated Ag_2_Te nanowires (PC-Ag_2_Te NWs); PANI, Polyaniline.).

## Data Availability

No new data were created or analyzed in this study. Data sharing is not applicable to this article.
